# Tissue-Specific Functional Networks for Prioritizing Phenotype and Disease Genes

**DOI:** 10.1371/journal.pcbi.1002694

**Published:** 2012-09-27

**Authors:** Yuanfang Guan, Dmitriy Gorenshteyn, Margit Burmeister, Aaron K. Wong, John C. Schimenti, Mary Ann Handel, Carol J. Bult, Matthew A. Hibbs, Olga G. Troyanskaya

**Affiliations:** 1 Department of Computational Medicine and Bioinformatics, University of Michigan, Ann Arbor, Michigan, United States of America; 2 Department of Internal Medicine, University of Michigan, Ann Arbor, Michigan, United States of America; 3 Lewis-Sigler Institute for Integrative Genomics, Princeton University, Princeton, New Jersey, United States of America; 4 Department of Molecular Biology, Princeton University, Princeton, New Jersey, United States of America; 5 Molecular & Behavioral Neuroscience Institution, Department of Psychiatry, and Department of Human Genetics, University of Michigan, Ann Arbor, Michigan, United States of America; 6 Department of Biomedical Sciences, College of Veterinary Medicine, Cornell University, Ithaca, New York, United States of America; 7 The Jackson Laboratory, Bar Harbor, Maine, United States of America; 8 Trinity University, Computer Science Department, San Antonio, Texas, United States of America; 9 Department of Computer Science, Princeton University, Princeton, New Jersey, United States of America; The Centre for Research and Technology, Hellas, Greece

## Abstract

Integrated analyses of functional genomics data have enormous potential for identifying phenotype-associated genes. Tissue-specificity is an important aspect of many genetic diseases, reflecting the potentially different roles of proteins and pathways in diverse cell lineages. Accounting for tissue specificity in global integration of functional genomics data is challenging, as “functionality” and “functional relationships” are often not resolved for specific tissue types. We address this challenge by generating tissue-specific functional networks, which can effectively represent the diversity of protein function for more accurate identification of phenotype-associated genes in the laboratory mouse. Specifically, we created 107 tissue-specific functional relationship networks through integration of genomic data utilizing knowledge of tissue-specific gene expression patterns. Cross-network comparison revealed significantly changed genes enriched for functions related to specific tissue development. We then utilized these tissue-specific networks to predict genes associated with different phenotypes. Our results demonstrate that prediction performance is significantly improved through using the tissue-specific networks as compared to the global functional network. We used a testis-specific functional relationship network to predict genes associated with male fertility and spermatogenesis phenotypes, and experimentally confirmed one top prediction, *Mbyl1*. We then focused on a less-common genetic disease, ataxia, and identified candidates uniquely predicted by the cerebellum network, which are supported by both literature and experimental evidence. Our systems-level, tissue-specific scheme advances over traditional global integration and analyses and establishes a prototype to address the tissue-specific effects of genetic perturbations, diseases and drugs.

## Author Summary

Tissue specificity is an important aspect of many genetic diseases, reflecting the potentially different roles of proteins and pathways in diverse cell lineages. We propose an effective strategy to model tissue-specific functional relationship networks in the laboratory mouse. We integrated large scale genomics datasets as well as low-throughput tissue-specific expression profiles to estimate the probability that two proteins are co-functioning in the tissue under study. These networks can accurately reflect the diversity of protein functions across different organs and tissue compartments. By computationally exploring the tissue-specific networks, we can accurately predict novel phenotype-related gene candidates. We experimentally confirmed a top candidate gene, *Mybl1*, to affect several male fertility phenotypes, predicted based on male-reproductive system-specific networks and we predicted candidates related to a rare genetic disease ataxia, which are supported by experimental and literature evidence. The above results demonstrate the power of modeling tissue-specific dynamics of co-functionality through computational approaches.

## Introduction

Phenotypes caused by mutations in genes often show tissue-specific pathology, despite organism-wide presence of the same mutation [1], [2], [3], [4]. Therefore, a logical genomics approach to infer candidate genes and their functions is to integrate large-scale data in a tissue-specific manner. However, such efforts are hampered by the lack of adequate tissue-specific training and feature data and by the methodologies to model tissue-specificity systematically in human or other mammalian model organisms.

Functional relationship networks, representing the likelihood that two proteins participate in the same biological process, provide invaluable information for phenotype gene discovery, pathway analysis, and drug discovery [5], [6], [7], [8], [9], [10], [11]. In human and model mammalian organisms, these networks have been used to predict genes associated with genetic diseases or phenotypes through computational mining of the network structure [5], [6], [7], [8], [10], [11]. For example, we have previously generated a mouse functional relationship network and used it to identify that *Timp2* and *Abcg8* are bone-mineral density (BMD)-related genes [11], though neither of these were previously detected in quantitative genetics studies. So far, these analyses have been limited to global functional networks representing the overall relationships between proteins without accounting for tissue specificity. Analyses based on global functional relationship networks, while effective, ignore a critical aspect of biology that could significantly improve their utility: genetic diseases often target specific tissue(s) and thus perturbations of proteins or pathways may have differential effects among diverse tissues. For example, *Timp2*, which we have previously identified to be related to BMD [11], is also involved in the control and/or development of neurodegenerative disease [12]. Such multi-functionality is not directly reflected by the global network but would be revealed by different connections in tissue-specific networks. Therefore, computational modeling and analyses of tissue-specific networks are needed to identify phenotype-associated genes that exhibit tissue-specific behavior.

Current approaches to create functional relationship networks are difficult to apply in a tissue-specific manner. Typically, networks are constructed by integrating data sources that vary in terms of measurement accuracy as well as biological relevance for predicting protein functions. Machine learning methods, such as Bayesian networks, learn the relative accuracy and relevance of datasets when given a ‘gold standard’ training set, which consists of gene pairs that are known to work in the same biological process. Then probabilistic models are constructed to weigh and integrate diverse datasets based on how accurately they recover the ‘gold standard’ set. The networks generated by this approach lack tissue-specificity information, because systematic collections of large-scale data or ‘gold standard’ pairs with quantitative tissue-specific information are often not available.

Here, we address the tissue-specificity challenge by simulating the natural biological mechanism that defines tissue-specificity: co-functionality in most cases would require the presence of both proteins in the same tissue. Inspired by our previous efforts to establish biological process-specific networks, such as networks specifically related to the cell cycle or to mitochondrial biogenesis [13], [14], [15], we integrate low-throughput, highly-reliable tissue-specific gene expression information (e.g. RT-PCR, *in situ* hybridization, *etc.*) from the Mouse Gene Expression Database (GXD) into our probabilistic framework when learning the reliability of each data source. Such an approach is more intuitive for the tissue-specific network setting because it is relatively less likely that a non-expressed gene would collaborate with an expressed gene even though they are ‘functionally related’ in the global sense (*i.e.* co-annotated to either a GO term or a KEGG pathway). There are exceptions to this guideline, including signaling and hormonal pathways that traverse multiple organ systems. However, many cellular processes important for phenotypes are largely restricted to specific tissues. Therefore, by constraining the ‘gold standard’ to pairs of genes that are both expressed in a tissue, we are able to establish functional networks that are highly specific in capturing the dynamic properties of different tissues.

In addition to generating the first tissue-specific networks for the laboratory mouse, we also explicitly tested the potential of using such networks to predict phenotype-associated genes. To do so, we mapped diverse phenotypes to their respective tissues in the laboratory mouse, according to the terminology and description of the phenotypes. We show that the tissue-specific functional relationship networks can improve our prediction accuracy for phenotype-associated genes compared to a single global functional relationship network through computational analyses, and through experimentally confirmed predictions of novel fertility-related genes and visualization of their local networks. We further identified candidate genes specifically predicted by the cerebellum network to be related to ataxia, which are supported by both literature and experimental evidence. Our networks are publicly available at http://mouseMAP.princeton.edu, which features the ability to compare networks across tissues for analyzing the dynamics of functional relationships. Our current framework covers 107 major tissues in the laboratory mouse and focuses on cross-network comparison and phenotype-associated gene discovery. However, as more data become available, this approach will serve as a prototype for applications to pathway analyses and drug screening.

## Results

In this study, we develop and apply a novel algorithm that generates tissue-specific functional networks in the laboratory mouse by integrating diverse functional genomic data, and we demonstrate that our tissue-specific networks are more accurate in predicting phenotype-related genes than a single global functional network. In the following sections, we first outline the strategy used to generate tissue-specific networks by interrogating gene expression profiles across tissues and integrating different data sources using Bayesian statistics. Second, we developed a cross-network comparison metric for identifying significantly changed genes across networks which are enriched in tissue specification and development. Third, we quantitatively demonstrate that combining our tissue-specific networks with a state-of-the-art machine learning algorithm can produce improved predictions of genotype-phenotype relationships compared to previous single global networks [11]. Fourth, we identify candidate genes related to male fertility specifically predicted by our tissue-specific networks (but not by the global network), and verify a top prediction in an independent, unbiased mutant screen. Finally, we used cerebellum-specific network to predict genes associated to a less-studied disease, ataxia, which are supported by both literature and experimental evidence. The predictions made by our approach for all examined networks are available online at http://mouseMAP.princeton.edu.

### Constructing a Bayesian model to establish tissue-specific functional networks

**Figure 1:**
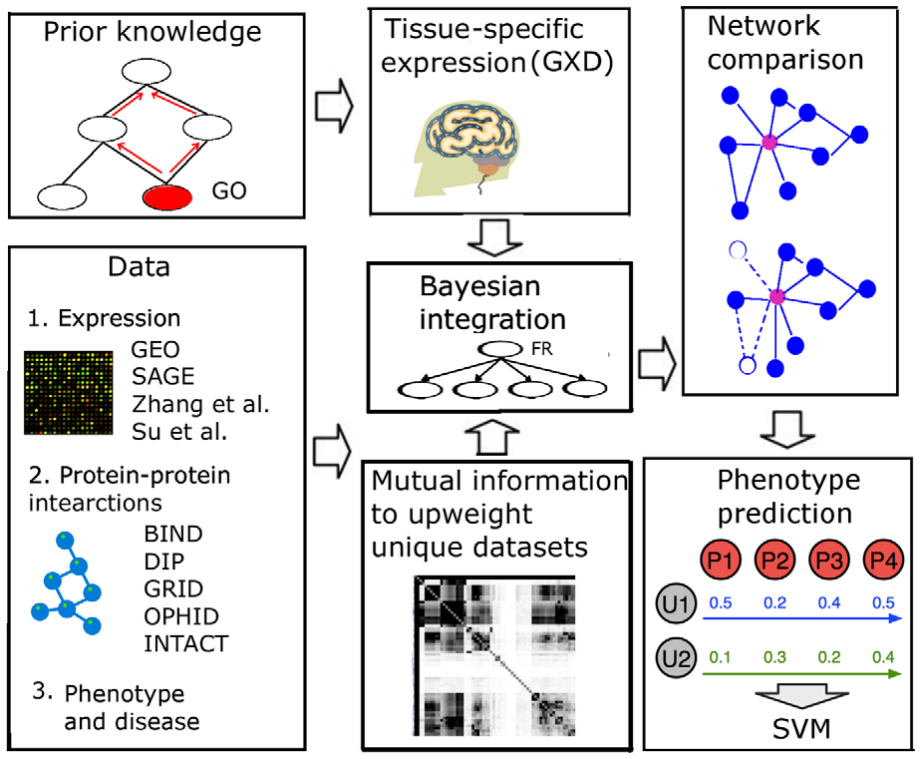
Strategy for constructing tissue-specific networks and predicting phenotype-associated genes. Diverse functional genomic datasets such as expression, protein-protein interactions and phenotype information were integrated in a Bayesian framework to generate tissue-specific networks. Input datasets were probabilistically “weighted” based on how informative they were in reflecting known co-functional proteins that are both expressed in a given tissue. To account for overlap in information in multiple datasets (especially the large number of gene expression microarray datasets), mutual information-based regularization was used to down-weight datasets showing significant overlap with each other. These networks were then used as input into a Support Vector Machine classifier to predict phenotype related genes. Finally, we implemented a web interface that allows network comparison between tissues.

A common mechanism resulting in tissue-specific protein functionality is the modulation of gene expression levels between tissues [16], [17], [18]. This observation is our theoretical foundation for establishing tissue-specific networks, in which links between proteins represent the probability that they are involved in the same biological processes within a specific tissue. To simulate such tissue-specificity, we developed a Bayesian approach (**Figure 1**) that incorporates highly-reliable, low-throughput measures of tissue-specific gene expression into training set, which we utilized to produce networks focused on the real functional relationships occurring within the tissue under consideration. This Bayesian framework essentially learns how informative each dataset is given a set of ‘gold standard’ training pairs, *i.e.* pairs of proteins known to be functional in the same biological process and both expressed in the tissue of interest.

In the global (non-tissue-specific) sense, following previous definitions [5], ‘gold standard positives’ are defined by co-annotation to specific Gene Ontology (GO) biological process terms [19], while ‘gold standard negatives’ are defined as pairs that both have specific GO annotations yet do not share any annotations. For each tissue-specific gold standard set, a positive pair has to meet two requirements: first, the pair must be ‘co-functional’ as defined in the global sense, and second, both genes must be expressed in the tissue under consideration as evident in highly reliable, low-throughput expression datasets, which, in most cases, is necessary for the pair to have a functional relationship in that tissue. These tissue-specific gold standards are then used to quantify how relevant each genomic dataset is in recovering tissue-specific functional relationships, regardless of the tissue of origin for each genomic dataset. This allows us to leverage the entire compendium of high-throughput genomic data to generate accurate tissue-specific networks, even for tissues which do not have existing tissue-specific whole-genome experiments, by relying on non-tissue-specific datasets, heterogeneous samples, and potentially related tissues and experiments. For example, biliary tract, which is not specifically represented in our current collection of high-throughput features used for classification, can still be accurately predicted by utilizing information from related, heterogeneous samples, such as gene expression microarrays of whole liver or the hepatic system, as well as non-tissue-specific information, such as sequence phylogeny and *in vitro* binding assays. Thus our approach can leverage the implicit relationships between a dataset and a tissue and therefore enables generation of tissue-specific networks even from feature data that is not resolved for a specific tissue type.

For tissue-specific expression information, our gold standards rely on the Gene Expression Database (GXD) of the Mouse Genome Informatics group (MGI). GXD provides an extensive, hierarchically structured dictionary of anatomical expression results for mouse to allow us to carry out our analysis [20]. The data in GXD are derived from traditional, “small-scale” expression experiments, such as *in situ* hybridization, RT-PCR, and immunohistochemistry, which simply reflect presence or absence of a gene within the tissue examined. No high-throughput expression data were used for our gold standard construction. In total, there are 107 tissues included in our analysis.

We pursue two main goals in this study: First, we generate tissue-specific networks that synthesize as much data as possible and provide these networks to the public through an online visualization interface at http://mouseMAP.princeton.edu. For this, we gathered diverse genomic data for mouse as inputs (**Dataset S1**) to support the functional relationships, including protein-protein physical interactions [21], [22], [23], [24], homologous functional relationship predictions from simpler organisms [9], phenotype and disease data [19], [25] and 960 expression datasets, totaling 13632 experimental conditions [26], [27], [28], [29]. The reliability of each dataset is learned through Bayesian network classifier training, using the tissue-specific gold standards described above. Essentially, a dataset deemed more relevant and accurate for the tissue under consideration will be given higher weight, and the final probability of pair-wise functional relationships is determined by updating the initial probability (prior) based on the weighted input of all genomic datasets. This procedure resulted in tissue-specific probabilistic functional relationship networks for the laboratory mouse that effectively summarize these diverse data sources and enable biology researchers to easily explore the resulting functional landscape. Second, we test the hypothesis that tissue-specific networks could assist us to predict phenotype-related genes more accurately. In this case, to prevent circularity in our methodology, phenotype and disease data were excluded from network generation, and the results were used to predict novel phenotype-associated candidate genes. We demonstrate that tissue-specific networks enhance biological clarity and result in more accurate predictions. Our resulting networks and predictions provide biology researchers with functional interactions specific to each tissue as well as phenotype hypotheses of genes.

### Robust recovery of tissue-specific functional relationships

One key application of tissue-specific networks is to identify novel genes and relationships between genes that may be specific to a particular tissue. To computationally evaluate our ability to identify novel relationships, we used cross-validation to test whether our tissue-specific Bayesian scheme is more accurate than the global network. Cross-validation was used to assess predictions by evaluating the accuracy of recovering subsets of known annotations withheld during the training process. Specifically, we performed 3-fold cross-validation, by holding out one third of the tissue-specific ‘gold standard’ pairs in each of the three iterations. We learned the parameters in the Bayesian networks, *i.e.*, the reliability of each dataset, through the other two thirds of the ‘gold standard’, and then used these networks to predict the probabilities for the held-out one third of the protein pairs.

**Figure 2:**
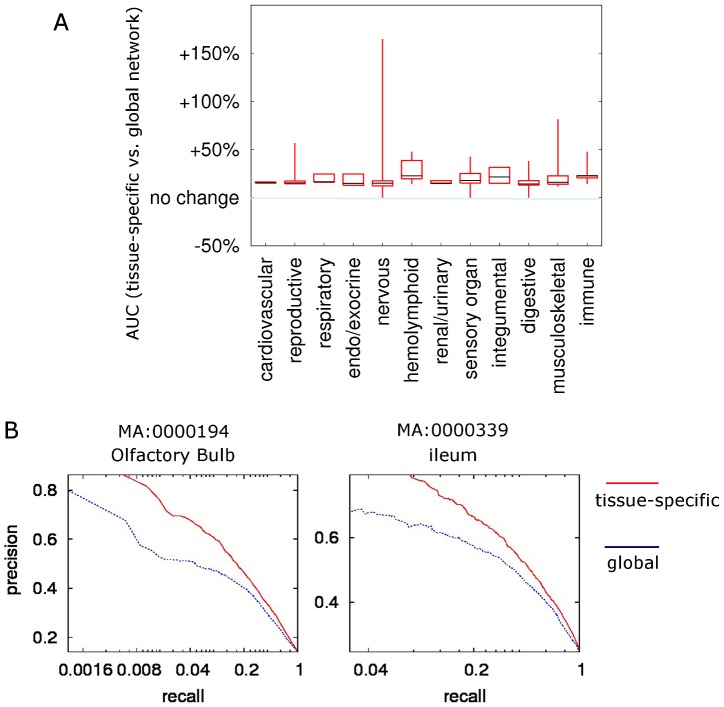
Tissue-specific networks are more accurate than the global network in reflecting protein functional relationships. **A.** 107 tissues were grouped into major body systems according to the anatomical hierarchical structure maintained in GXD [20]. Through three-fold cross-validation, the performance of tissue-specific networks was compared against the global network and the percentage improvement of tissue-specific networks over the global network was plotted. All tissue-specific networks out-performed the global network in this cross-validation analysis. Improvements were consistent across tissues belonging to all major organ systems. Candle-stick plots (minimum, 25%, median, 75% and maximum) represent the distribution of percentage AUC improvement for all tissues in a specific system. **B.** Example precision recall curves of tissue-specific and the global network, generated using three-fold cross-validation. Across the entire precision-recall space, tissue-specific networks performed better than the global network. Complete precision-recall figures for all networks are included in **Dataset S2**.

Compared to a single global functional relationship network, our approach significantly improved our ability to predict tissue-specific functional linkages. The mean AUC (area under the receiver operating characteristic curve, which represents the accuracy in recovering tissue-specific functional relationships) for the global network estimated through three-fold cross-validation was 0.68. Tissue-specific networks achieved median AUC of 0.72. With a random baseline of 0.5 in AUC, this represents a ∼20% improvement of the tissue-specific networks over the predictive power of the global network. This improvement is consistent over all 12 major organ systems defined by GXD [20]. (**Figure 2A**). Immune system-related networks acquired the most median improvement of 22.7% and digestive system-related networks achieved least median improvement of 14.3%. For example, for lymphoid system (MA:0002435), we improved our AUC from 0.65 to 0.72 and for ventricular zone, brain, we improved from 0.65 to 0.77. Such improvement is consistent across the entire precision-recall spaces (**Figure 2B**, **Dataset S2** for all precision-recall curves). In all cases, tissue-specific networks performed better than the global network in predicting functional relationships specific to that tissue, which demonstrates the robustness of our integration approach across different systems and tissues in the laboratory mouse.

### Network comparison reveals altered gene-gene functional connections across tissues enriched for activated biological processes

**Figure 3:**
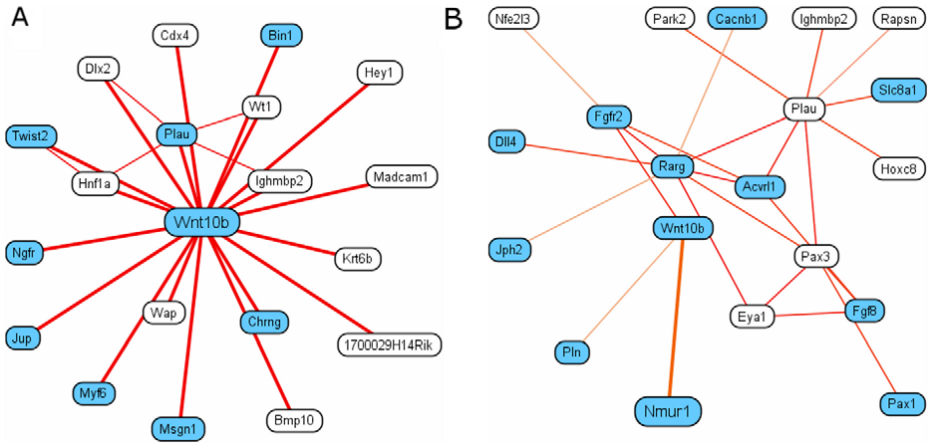
Top connected genes of Wnt10b in muscle-specific and bone-specific networks. In A, blue-highlighted genes are directly involved in skeletal muscle development. In B, blue-highlighted genes are involved in bone minerization or bone structure formation. The enrichment of genes involved in the above processes reflects the differential roles of Wnt10b in skeletal muscle and bone.

One important application of our tissue-specific networks is to identify functional relationships between genes that change significantly across tissues. This provides a platform for analyzing tissue-specific molecular interactions, as well as tissue-specific roles for genes that are ubiquitously expressed but play different roles in different tissues. For example, Wnt10b (wingless related MMTV integration site 10b) is expressed in many tissues throughout development and participates in many biological processes including bone trabecular formation [30] and cell differentiation involved in skeletal muscle development [31]. The interactors of Wnt10b in our muscle-specific and bone-specific functional networks reflect its differential roles in these two tissues. The top neighbors in the muscle-specific network consist of genes responsible for skeletal muscle development (**Figure 3A**). For example, BIN1 participate in the biological process muscle cell differentiation (GO:0042692) [32], PLAU is involved in the process skeletal muscle tissue regeneration (GO:0043403) in rat and MYF6 directly function in muscle cell biogenesis [33]. In fact, 8 out of the 19 top connected nodes of Wnt10b in the muscle-specific network are involved in skeletal muscle cell development, reflecting the functional role of Wnt6b. On the contrary, in the bone-specific functional network, the top neighbors of Wnt10b consist of genes involved in bone mineralization and bone structure formation (**Figure 3B**), representing 12 out of the 19 top connected nodes. This observation suggests that our networks can provide a resource for comparing the dynamic functions of a single gene across different tissues.

To quantify gene connectivity changes across networks, we developed a metric that captures how much the edges involving a gene differ across networks (see methods), and we implemented a web-based visualization interface (http://mouseMAP.princeton.edu) allowing users to query genes of interest and compare the local network between tissues. Essentially, connectivity change of a gene is defined by the sum of absolute values of fold changes (over prior) of connections between this gene to all other genes. Some genes vary greatly in their connectivity between tissues, potentially reflecting their tissue-specific roles. Of the top 100 altered genes, they were significantly enriched for “anatomical structure development” (GO:0048856) and “organ development” (GO:0048513). Additionally, genes with connectivity altered in specific tissues compared to the global network, tend to be enriched for GO terms related to the tissue under consideration. For example, when comparing the nervous system-specific network (MA:0000016) against the global network, the top changed genes are enriched in “central nervous system development” (GO:0007417), “diencephalon development” (GO:0021536), and “brain development” (GO:0007420) (**Table 1**). The full enrichment analysis is provided in **Dataset S3**.

**Table 1:** Example enriched Gene Ontology terms in the tissue MA:0000016 nervous system.

GO:0048856	anatomical structure development	3.40E−09
GO:0007417	central nervous system development	4.27E−09
GO:0048513	organ development	2.61E−08
GO:0021536	diencephalon development	5.25E−07
GO:0021984	adenohypophysis development	8.94E−07
GO:0007420	brain development	2.18E−06
GO:0048732	gland development	1.48E−05
GO:0032502	developmental process	1.62E−05
GO:0030900	forebrain development	2.10E−05
GO:0007399	nervous system development	2.27E−05

### Prioritizing phenotype-associated genes in relevant tissue-specific networks

A key hypothesis in this study is that analyzing tissue-specific networks may improve our ability to identify phenotype-related genes. To test this hypothesis, we regenerated tissue-specific networks using the same Bayesian approach as above, but excluded all phenotype and disease data as inputs to avoid circularity in our cross-validations. Then, we mapped 451 phenotypes to their most related tissue in the laboratory mouse according to the terminology and description of these phenotypes in the Mammalian Phenotype ontology [19]. For each phenotype, we compared novel predictions made using the appropriate tissue-specific network as compared to using the global network. This method is based on our previously developed machine learning scheme (network-based SVM) [11] that mines information in functional relationship networks to prioritize candidate genes according to their links to known genes related to a disease or phenotype.

**Figure 4:**
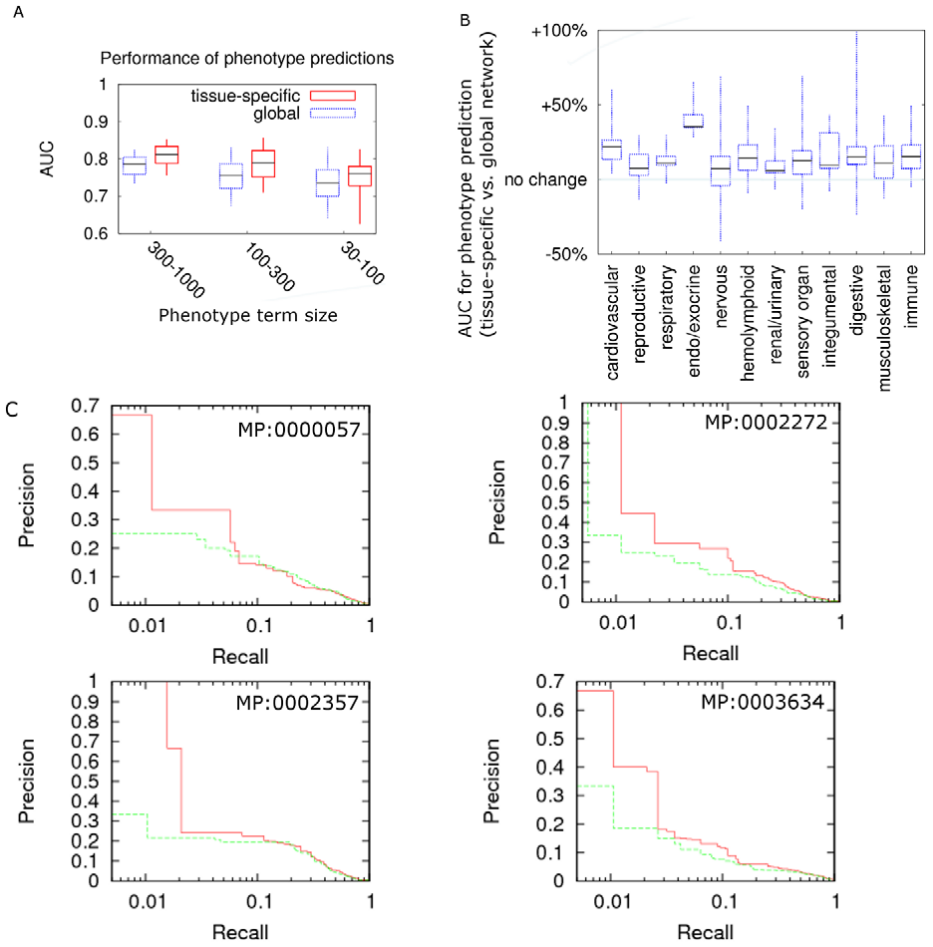
Tissue-specific networks perform better than the global network in predicting genes related to different phenotypes. By mapping phenotypes to different tissues according to their terminology and description, we are able to compare the performance of tissue-specific networks and the global network in predicting phenotype-related genes. Candle-stick plots (minimum, 25%, median, 75% and maximum) show the distribution of percentage AUC improvement when predicting phenotype-related genes. **A.** Phenotypes were grouped according to the number of annotated genes. Tissue-specific functional networks show consistent improvement across different phenotype sizes. **B.** Phenotypes were grouped according to major organ systems of their corresponding tissue. Improvements were consistent across all major systems. **C.** Example precision-recall curves for “abnormal osteogenesis” (MP:0000057), “abnormal nervous system electrophysiology” (MP:0002272), “abnormal spleen white pulp morphology” (MP:0002357), and “abnormal CNS glial cell morphology” (MP:0003634) using both tissue-specific networks (shown in red) and global networks (shown in green). For phenotypes such as these, tissue-specific networks are necessary to make accurate predictions.

To test whether our tissue-specific networks are more capable of identifying phenotype-associated genes than the global network, we used bootstrap bagging [34] to evaluate which network performs better. Bootstrap bagging is suitable for phenotype predictions, where positive examples (known phenotype-associated genes) and negative examples (random genes) are highly imbalanced [35]. Its stability and comparably good performance in estimating error rates has been tested in extensive simulations for positive example set sizes ranging from less than 20 [35] to >200 [36], which is the approximate range we are using in our evaluation. For the 451 mapped phenotypes, the median AUC when utilizing tissue-specific networks is 0.794, representing an improvement of 11.8% over utilizing the global functional network. For many phenotypes, using tissue-specific networks can improve our ability to extract potentially experimentally-verifiable predictions. For example, at one percent recall (the low recall end is where most of the follow-up experimental confirmations will focus on), we achieved a precision of 1.00 compared to 0.33 using global network for the phenotype abnormal spleen white pulp morphology (MP:0002357), and a precision of 0.5 compared to 0.28 for abnormal malpighian tuft morphology (MP:0005325). Additionally, the AUC for “abnormal osteogenesis” (MP:0000057) was 0.77 using the global network, but 0.81 for tissue-specific networks. The AUC for “abnormal nervous system electrophysiology” (MP:0002272) using the global network was 0.716, but was 0.763 using the nervous system-specific network (**Figure 4C** for example precision-recall curves). Such significant improvement demonstrates the potential of mining tissue-specific networks to prioritize phenotype-associated genes.

Performance improvements were consistent across phenotypes of different sizes (**Figure 4A**). For phenotypes with 300–1000 annotated genes (around 1.5% to 5% of genome), we achieved a median AUC of 0.814 (improvement of 8.7%); for phenotypes with 100–300 genes, the median AUC was 0.792 (improvement of 13.0%); and for phenotypes with 30–100 genes, the median AUC was 0.769 (improvement of 11.0%). At 10 percent recall for the 300–1000, 100–300, and 30–100 groups, we achieved precisions of 14.8, 17, and 20 fold over random, respectively. This consistency indicates the robustness of tissue-specific networks against the number of known genes in predicting phenotype-associated genes.

Performance improvements were also consistent across different major organ systems. Phenotypes involved in the endo/exocrine system achieved the most significant improvement in AUC (+35%, compared to global networks against baseline of 0.5) and those in cardiovascular system achieved 21.8% improvement in AUC. However, prediction accuracy was improved across all major systems, with the least improvement of 5.9% in renal/urinary phenotypes. Phenotypes related to musculoskeletal systems achieved the highest AUC of 0.82 and the group with lowest AUC was digestive system, which still achieved an average of 0.78. The consistency in improvements across different organ systems demonstrates the robustness of our modeling framework to predict phenotype-related genes in a tissue-specific manner.

### Predicting and testing phenotype/disease genes using the tissue-specific networks

We focused on two cases to illustrate how our tissue-specific networks can facilitate disease gene discovery. These two phenotypes represent two extremes of the phenotype/disease-associated gene prediction problem. The first, reduced male fertility, is a broadly defined, common phenotype with many causative genes already known. The second, ataxia, is a rare neurological disorder affecting ∼3–10/100,000 of the general population [37], [38], [39]. Roughly 40 genes are known to be associated with this disease, but the majority of both familial and sporadic cases remain unexplained. Predicting candidate genes related to rare genetic diseases is challenging in that little prior knowledge is available for these diseases. These phenotypes are related to two different tissue-categories (reproductive and neurological systems), enabling us to highlight the broad applications of our approach across organ systems. We used these two examples and experimental confirmations to demonstrate the power of tissue-specific networks to discover disease genes.

First, we used male fertility related phenotypes to test the performance of tissue-specific networks to predict phenotype-related genes. To do so, we utilized a recent, nearly comprehensive literature review of genes involved in mammalian spermatogenesis and male fertility phenotypes [40], which we organized into a hierarchy of male fertility-related phenotypes (**Dataset S4**). This curation effort is independent of, and more comprehensive than, the current GO or MP annotations related to male fertility, which makes these lists excellent, non-circular test sets. We tested whether the testis-specific network could predict male fertility genes more robustly than the globally integrated network, and found that the testis-specific network significantly improved our ability to predict spermatogenesis-related phenotypes. For example, for predicting genes related to ‘spermatid head and nuclear modifications,’ we achieved 4.5-fold improvement in precision at 1 percent recall; for ‘acrosome-related genes,’ we achieved 3.6-fold improvement; and for ‘germ/Sertoli cell interaction genes,’ we achieved 3.3-fold improvement. On the other hand, for terms that are not specifically related to male-reproductive systems, such as ‘association with methylation and acetylation,’ and ‘association with Golgi Apparatus,’ we observe no performance improvements using the testis-specific network. This illustrates that tissue-specific functional relationship networks are tuned to predict phenotypes closely related to these tissues.

**Figure 5:**
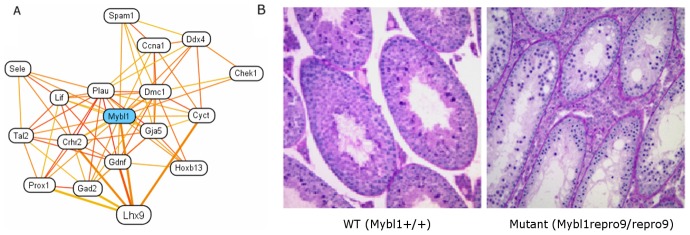
Prediction and verification of infertility-related genes through male reproductive system-specific networks. **A.** Local functional relationship network of the gene *Mybl1* in the male reproductive system. The top 18 genes connected to the query set with connection weights higher than 0.634 are displayed. These top functionally related proteins include well characterized male infertility genes such as *Dmc1*, *Ddx4*, and *Cyct*. **B.** Histological cross-sections of oval seminiferous tubules show that wild type (*Mybl1^+/+^*) testis tubules contain many developing germ cells, while mutant (*Mybl1^repro9/repro9^*) testis tubules contain many fewer germ cells and more empty space, indicative of infertility.

We selected *Mybl1* to demonstrate the specific utility of the male-reproductive network to predict fertility related genes. *Mybl1* (MGI:99925) is among our top candidates in multiple phenotypes related to male fertility, including ‘association with chromatoid body and manchette’, ‘transcription factor involved in spermatogenesis’ and, ‘spermatogenesis’. However, in the global network, *Mybl1* was not a strong candidate for these phenotypes, as it was predicted with negative values. Therefore *Mybl1* is an ideal candidate to test the accuracy of our tissue-specific network-based phenotype predictions. In our male-reproductive network, the majority of the top interactors of *Mybl1* are indeed well-known male fertility genes (**Figure 5A**), including *Dmc1* (required for meiosis and male fertility [41]), *Ddx4* (a DEAD-box helicase required for male, but not female, germ cell development [42]), *Cyct* (encoding testis-specific cytochrome c [43]) and *Lhx9* (a LIM homeobox required for sex differentiation and normal fertility [44]). Moreover, *Mybl1* was independently identified recently in an unbiased mutagenesis screen for infertility phenotypes involving meiotic arrest [45]. We found that the *Mybl1* mutants are characterized by low testis weight and depletion of male germ cells, as shown in **Figure 5B**. Additionally, analysis of the mutant testis transcriptome suggested that MYBL1 is a “master regulator” of the meiotic cell cycle and transcriptional program [46], and at least one gene regulated by MYBL1, *Cyct*, is among the top interactors of MYBL1 predicted by our network. Together, these findings on an infertility phenotype and suggestions of a corresponding potential mechanism confirm the accuracy of predictions from our tissue-specific network and show that when taken with expression analyses and other data, they can be used as a basis for functional testing.

In addition to the well-studied phenotype of male infertility, we also examined a less well-understood disease, ataxia, to investigate whether our tissue-specific networks can identify genes related to phenotypes or diseases with limited prior knowledge. Gene identification through genetic approaches, such as pedigree analyses, has had a major impact on our understanding of ataxia (over 40 candidate genes identified so far). Genetic testing is now an integral part of assessment. Routinely, a blood sample of any new ataxia case is mailed in for laboratory evaluation. However, the majority of the sporadic cases as well as the familial cases are so far unexplained. We curated the known gene list (43 in total) related to human ataxia, mapped these genes to their mouse orthologs, and used this list as seeds to predict additional candidate genes using our cerebellum-specific network, which is the major tissue affected by ataxia.

**Figure 6:**
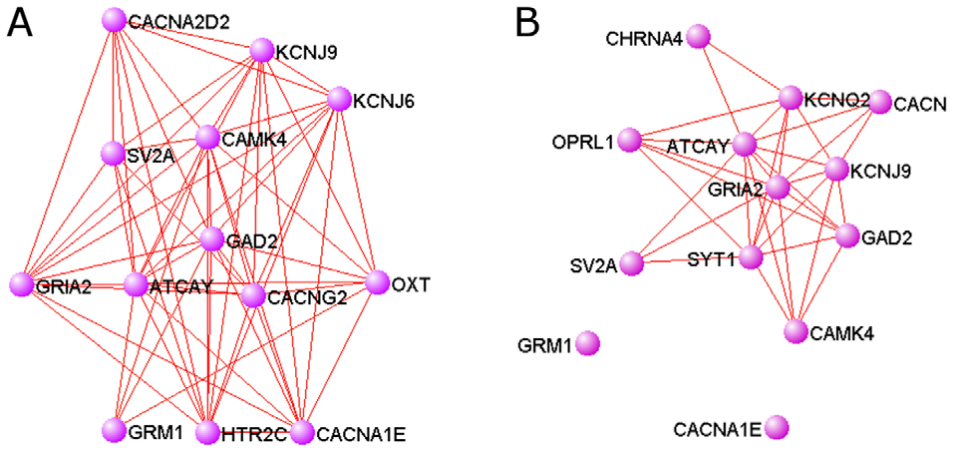
Top connected genes to *Atcay* in the cerebellum-specific network reveals likely ataxia candidates. Edges with weight greater than 0.9 are shown. In the cerebellum network (**A**), *Grm1* and *Cacn1a* are the top predicted connections to *Atcay*, with confidences of 0.902 and 0.943, respectively. Both genes are closely connected to *Atcay* and its top 10 neighbors. In the global network (**B**), *Grm1* and *Cacn1a* are much more weakly connected to *Atcay* (0.763 and 0.647, respectively), and are not identified as top connectors to *Atcay*. *Grm1* and Cacn1a are not connected to *Atcay* or any of its top 10 neighbors in the global network.

Our cerebellum-specific network reveals connections of ataxia-related genes not shown in the global network. A key, known ataxia gene is *Atcay* (ataxia, cerebellar, Cayman type homolog (human)), and in the cerebellum-specific network, two of its top interactors are *Cacna1e* (with connection confidence 0.943, ranked 18) and *Grm1* (0.902, ranked 46) (**Figure 6**). These are plausible candidate genes since *Grm1* is a known mouse ataxia gene [47], and *Cacna1e* encodes a subunit of an R-type calcium channel, while mutations to the related protein family member *Cacna1a*, encoding a subunit of an L-type calcium channel, causing spinocerebellar ataxia. However, in the global network these interactions are much weaker (0.647 for *Cacna1e* and 0.763 for *Grm1* respectively), and would not be identified in the top 100 connections of *Atcay*, which supports the utility of tissue-specific networks relevant to ataxia to identify candidate genes (**Figure 6B**).

In addition to identifying these novel, likely correct edges, we also identified novel candidates using our SVM-based approach described above. Out of our top 10 novel candidates, we found strong evidence in the literature for 4 of these genes to be associated to ataxia (**Table 2**); suggesting at least a 40% success rate at low levels of recall. Among these, SORBS1 physically interacts with ATXN7, an autosomal dominant gene causing cerebellar ataxia [48]. RBFOX1 physically interacts with the c-terminus of ATXN2, another autosomal dominant gene causing cerebellar ataxia [48], [49]. It is thought that RBFOX1 might contribute to the restricted pathology of spinocerebellar ataxia type 2 (SCA2) [50]. The homozygous mouse knockout of a third gene, *Plcb4* induces ataxia, although no human patients have been identified with mutations in this gene. A fourth gene, *Plp1*, is implicated in Spastic paraplegia-2 and Pelizaeus-Merzbacher diseases [51], which are disorders closely related to ataxia. It is also a homologue of *Pmp22*, which is involved in Charcot Marie Tooth disease type 1A, a sensory neuropathy common in some forms of ataxia [52]. Thus, even in the case of less well-studied phenotypes or diseases, our tissue-specific approach is able to identify likely candidates as evidenced by our success rate of at least 40% for ataxia-related predictions based on the cerebellar network, compared to a background detection rate of less than 1/500.

**Table 2:** Evidence for top 10 predictions for ataxia-causing genes using mouse cerebellum-specific networks.

Gene	Rank in cerebellum network	Rank in global network	Evidence
PDK2	1	71	None
RBFOX1	2	9	Physical interaction with ATXN2
HLF	3	302	None
APBB1	4	241	None
PLCB4	5	84	Double knockout confirmed in mice
LRRC2	6	1778	None
TXLNB	7	356	None
SORBS1	8	743	Physical interaction with ATXN7
CYP2D6	9	476	None
PLP1	10	87	homologue of PMP22, implicated in ataxia-related Spastic paraplegia-2 and Pelizaeus-Merzbacher disease

## Discussion

Genetic diseases often manifest tissue-specific pathologies [1], [2], [3], [4]. Therefore, acquiring tissue-specific functional information is essential for biomarker identification, diagnosis, and drug discovery. Current integrative functional genomics approaches to study diseases or phenotypes generally do not analyze them in the context of specific tissues. Our work represents a conceptual advance to address tissue-specificity in genome-scale functional studies of phenotypes. We describe a strategy to systematically generate tissue-specific functional networks that are robust and accurate for mining phenotype-related genes, demonstrating the importance of tissue-specific approaches for understanding human diseases.

Our approach addresses the twin challenges of incomplete systematic knowledge of tissue-specific protein functions and of limited availability and coverage of tissue-specific high-throughput functional data. Due to this lack of systematically defined tissue-specific genomic data, our approach uses highly reliable, low-throughput measures of gene expression to constrain our gold standard examples into tissue-specific sets. As more tissue-specific protein functions are defined systematically, perhaps with the help of hypotheses generated by approaches such as this, tissue-specific functional interactions will be directly used for experimental testing. Many genomic datasets, especially physical interaction studies, such as yeast 2-hybrid screens, and large-scale genetic screens, utilize artificial or *in vitro* contexts that may or may not reflect tissue-specific functional roles. Other data, however, such as high-throughput gene expression datasets (*e.g.* microarrays or RNA-seq), is often collected in a specific tissue or cellular context and may thus reflect a more restricted, tissue-specific set of genes or proteins. In our approach, we use the power of Bayesian machine learning to learn the predictive power of each dataset, whether *in vivo* or *in vitro*, by utilizing training sets restricted to gene pairs that are both expressed in the same tissue or context. In this way, data from empirically relevant contexts are trusted, while irrelevant data are disregarded.

While our current study focuses on predicting genotype-phenotype associations using tissue-specific functional relationship networks, the potential application of tissue-specific networks extends far beyond predicting phenotype-associated genes. For example, just as perturbations of the same gene may lead to different phenotypic outcomes across different tissues; treatments with bioactive chemicals or drugs may manifest differential effects across different tissues. Our broad conceptual framework of utilizing tissue-specific expression to refine a global network could be brought into these application domains such as drug target identification.

## Methods

### Functional network construction through Bayesian integration

#### Basic framework

Our framework to generate both global and tissue-specific functional networks is based on naïve Bayesian network data integration coupled with mutual information-based regularization. Genomic datasets are weighted differentially based on how well they recover gold standard positive pairs in either global or tissue-specific training sets (see the section below for construction of tissue-specific gold standards). Specifically, we computed the posterior probability of a functional relationship given all available evidence following the scheme in [5]:
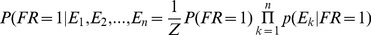
(1)Where *FR* = 1 represents that a pair of proteins is functionally related, *E_k_* represents the score for this pair in dataset *k*, and *Z* is a normalization factor. Intuitively, this probability *FR_ij_* for two proteins *i* and *j* represents how likely, given existing data and their accuracy and coverage, proteins *i* and *j* participate in the same biological process.

The assumption of conditional independence is a major factor penalizing the performance of naïve Bayesian integration, given that many biological datasets share information. We used multiple strategies to minimize the impact of information overlap based on the nature of the data integrated. For physical and genetic interaction datasets, we combined different data sources as described in the next section. For microarray gene expression data, which are the major data sources contributing to overlapping information, we regularize the contribution of each microarray dataset according to:
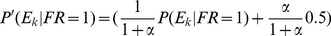
(2)Where 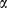 represents the ratio of the sum of mutual information between *k* and all other datasets to the entropy of *k*. Mutual information *I(X,Y)*, between a dataset *X* to *Y* is calculated according to:
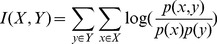
(3)The entropy of that dataset, *H(X)*, is calculated according to:
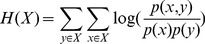
(4)


#### Gold Standard Construction

To learn the parameters in this Bayesian framework, we first established a gold standard that approximates a true set of functionally related proteins. We obtained Gene Ontology (GO) biological process branch annotations from the Mouse Genome Informatics database (MGI) [53]. Gold standard positives are defined as pairs that are co-annotated to a specific biological process GO term (with less than two hundred genes annotated to it) and negatives as those in which both members of the pair have specific annotations but do not share any.

For each tissue-specific Bayesian framework, we created a tissue-specific gold standard restricted to protein pairs that are both expressed in the tissue of interest, which approximates a set of functionally related proteins within a specific tissue context. We utilized the mouse Gene eXpression Database (GXD) [20] to determine which genes or proteins are expressed in each mouse tissue. The GXD data is based on traditional, low-throughput assays of gene expression localization, such as *in situ* hybridization, immunohistochemistry, and RT-PCR. Thus, these data are independent of, and more reliable than, the high-throughput expression data used as features for data integration. We selected 107 tissues with most annotated expressed proteins in GXD to study. For each specific tissue, the global gold standard positives are intersected with pairs that both express in the tissue; the gold standard negatives are restricted to pairs both expressing in the tissue but not functionally related.

#### Genome-scale data retrieval and pre-processing for generating networks

We collected diverse functional genomics data to use as input for the integration. All data used in phenotype analysis were acquired as of Jan 2011. All data were processed into pair-wise similarity scores *S(i,j)*, which reflect the similarity between proteins *i* and *j*:

Protein physical interactions: We acquired protein-protein physical interaction data from MiMI (Michigan Molecular Interactions) [54], BIND [21], BioGRID [24], DIP [55], IntAct [22], IPI, MINT [23] and Reactome [56]. This included 76749 interactions. These interactions are grouped by interaction and experiment type, such as affinity capture, two-hybrid, indirect complex, or co-purification. Each pair may be involved in multiple, different interaction or experiment types. Protein pairs of small experiment types (less than 1000 pairs) are grouped together, so that we have enough examples to learn probabilities in our Bayesian framework. These groups represent six binary datasets, representing the presence/absence of evidence for a physical interaction between a pair of proteins (Complete description of the grouped datasets is included in Dataset S1).

Expression data: To utilize the signals represented by diverse microarray data, we acquired mouse microarray datasets from GEO (977 datasets, 960 of them have more than or equal to three samples, totaling 13632 arrays) [29]. For each dataset, we calculated the Pearson correlation coefficient, *ρ*, to assess levels of co-expression between pairs of genes. The correlation coefficients were Fisher *z*-transformed [57] and normalized to *∼N(0,1)* to ensure normal distribution of datasets and comparability across different datasets and platforms, as previously described [58], [59].

Homologous functional relationship predictions: Previous analysis indicates that homologous functional relationships in simpler model organisms are a good indicator of functional relationship in higher model organisms [5]. We acquired the yeast functional network from [9] and mapped proteins and relationships to their corresponding laboratory mouse ortholog using InParanoid [60]. A single average score was taken in the case of multiple mappings.

Phenotype and disease: We acquired data from MGI [19] and the Online Mendelian Inheritance in Man (OMIM) database [25] annotations (mapped to orthologous mouse genes using InParanoid [60]). The similarity score (*S*) for the protein pair *i*, *j* of the phenotype and disease data is given by:
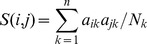
(5)Where *a_ik_* = 1 if protein *i* has phenotype *k* and *a_ik_* = 0 otherwise, and *N_k_* is the number of proteins involved in this phenotype/disease; and *n* is the total number of phenotypes and diseases. In this way, co-occurrence of phenotypes or diseases with less annotated genes will be given more weight than well-studied, broadly-defined phenotypes.

Phenotype and disease data are included in the networks displayed on our web interface, but were excluded from the networks used to predict phenotype-related genes to prevent circularity.

The above data are integrated together using the Bayesian framework (formulas 1–4) to generate both global and tissue-specific networks. The evaluation of each of the input datasets against each tissue-specific gold standard is included in **Dataset S5**.

#### Cross-validation of network performance

We used three-fold cross-validation to evaluate the performance of our tissue-specific networks and the global network. Each gold standard set was randomly partitioned into three subsamples. Each subsample was retained as a validation set while the other two were used for training the Bayesian networks. The AUC of the tissue-specific networks in recovering the held-out set was compared against that of the global network.

### Network-based phenotype gene prediction

#### Mapping between phenotypes and tissues

Phenotypes were mapped to tissues based on sub-string matches between phenotype and tissue descriptions. For example, the phenotype thyroid gland hyperplasia (MP:0003498) can be mapped to the tissue thyroid gland (MA:0000129). This resulted in 451 phenotype-tissue matches. In the rare case where multiple tissues mapped to a single phenotype, the network with the highest cross-validation performance was selected for evaluation.

#### Network-based phenotype gene prediction

We downloaded the mammalian phenotype (MP) ontology and annotations for mouse from MGI on May 4, 2011, including 196190 entries for 13438 genes in total. All annotations were propagated along the ontology hierarchy. If any allele of a gene was annotated to phenotype under consideration or a descendent of this phenotype term, we associated that gene with this phenotype. We then adopted the network-based candidate gene prediction scheme from [11]. Essentially, the connection weights from the integrated network to all positive examples (*i.e.* genes already known to be related to a phenotype) are utilized as features for linear support vector machine [61] classification:
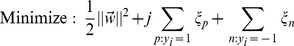
(6)

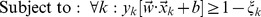
(7)where *x_k_* represents the connection weights to the positive examples, *y* equals to 1 or −1 depending on whether a gene is annotated to the phenotype term or not, *p* is any gene annotated to the term in study, and *n* is any of the other genes. *k* represents all training genes. *j* represents the relative cost of a wrongly classified positive example to a wrongly classified negative example.

#### Bootstrap bagging

We applied a unified scheme for evaluation and prediction based on bootstrapping, where examples were randomly sampled with replacement (0.632 bootstrap, that is, the expected fraction of selected data points is 0.632) [34]. For each bootstrap sample, a model was learned based on the selected examples, and the resulting classifier was used to create predictions for both un-selected (out-of-bag) examples and the unknown examples. The final classifier outputs were taken as the median of out-of-bag values across bootstraps for the training set (bootstrap cross-validation; evaluation), and as the median of all values across bootstraps for the unknown examples (bagging; prediction).

### Cross-network comparison

For each pair of networks, we quantify how much each gene has changed in the network relative to its neighbors. Suppose in network *X*, the connection weight between gene *i* and gene *j* is *X_ij_*, and in network *Y*, the connection weight between *i* and *j* is *Y_ij_*. The prior for network *X* is *P_x_*, and the prior for network *Y* is *P_y_*. The score representing how much the gene *i* changed from network *X* to network *Y* is defined as:
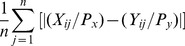
(8)
*n* represents all other genes. We then calculate the gene ontology enrichment of top 100 changed genes for each network against the global network using established techniques [62].

### Experimental validation

To examine the role of Myb1 in spermatogenesis, as described in detail elsewhere [46], novel ENU-induced fertility mutations were identified in a 3-generation breeding scheme. Standard histological methods were used for preliminary characterization of the *Mybl1* mutant phenotype.

We curated 43 genes causing ataxia that have been confirmed in human pedigree studies. These 43 genes were mapped to mouse one-to-one orthologs using the orthology defined by MGI [19], and were used as seeding genes for predicting additional candidates.

### Implementation

To allow dynamic visualization and cross-network comparison of our integration results, we developed the mouseMAP software (http://mouseMAP.princeton.edu), based on the open-source viewing framework Graphle that we developed in [63]. MouseMAP is based on the Prefuse Java visualization library, the Args4j command line parsing tool, and the SQLiteJDBC SQLite database driver. The basic functionality of mouseMAP allows querying one or multiple genes and retrieving the local network surrounding the query, with user-variable node number and confidence level cutoffs.

Our public, web-based system features cross-comparison of different networks that highlights connections in the newly queried network *vs.* the previously queried network, which allows us to compare the connections between different tissues of the same query gene(s). Gene information, including annotation, phenotype and disease association is retrievable through the interface. To facilitate general public use, mouseMAP also dynamically generates figure descriptions based on the current query and network structure.

## Supporting Information

Dataset S1 List of genomics datasets used in integration.
(XLS)

Dataset S2 Precision-recall figures for each tissue-specific network (red) versus the global (blue) network.
(ZIP)

Dataset S3 Functional enrichment of top 100 changed genes for each tissue-specific network.
(ZIP)

Dataset S4 Expert-created ontology of spermatogenesis-related phenotype terms.
(XLSX)

Dataset S5 The AUC of each expression dataset evaluated against each tissue-specific gold standard.
(ZIP)
