# Supplementary figures and images for: Tissue-Specific Functional Networks for Prioritizing Phenotype and Disease Genes (part 2 of 2)
Source: PLoS Comput Biol. 2012 Sep 27;8(9):e1002694. doi: 10.1371/journal.pcbi.1002694 (PMC3459891; doi:10.1371/journal.pcbi.1002694)

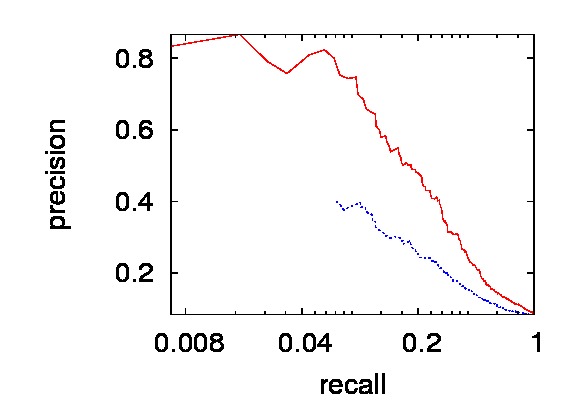

Supplement: Dataset S2 — Precision-recall figures for each tissue-specific network (red) versus the global (blue) network. (ZIP) [file pcbi.1002694.s002.zip › individual_figure/muscle.txt.jpg]

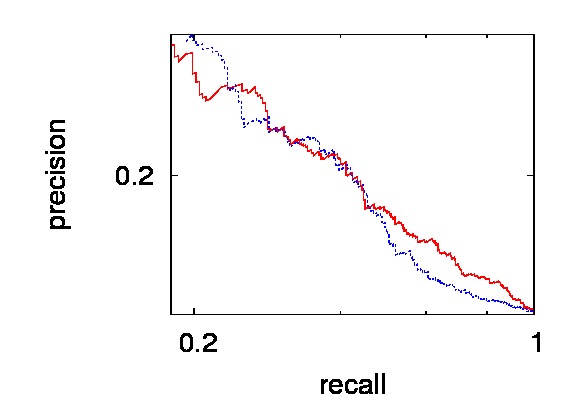

Supplement: Dataset S2 — Precision-recall figures for each tissue-specific network (red) versus the global (blue) network. (ZIP) [file pcbi.1002694.s002.zip › individual_figure/olfactory_epithelium.txt.jpg]

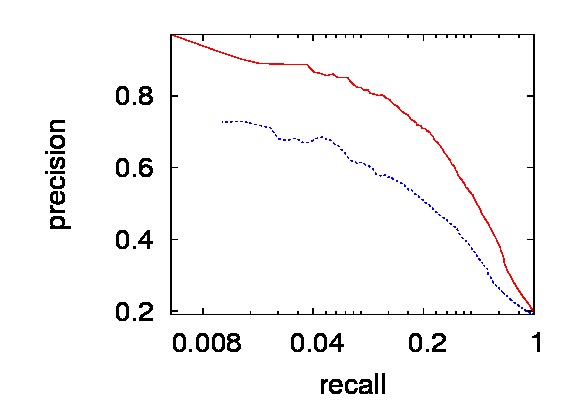

Supplement: Dataset S2 — Precision-recall figures for each tissue-specific network (red) versus the global (blue) network. (ZIP) [file pcbi.1002694.s002.zip › individual_figure/oocyte.txt.jpg]

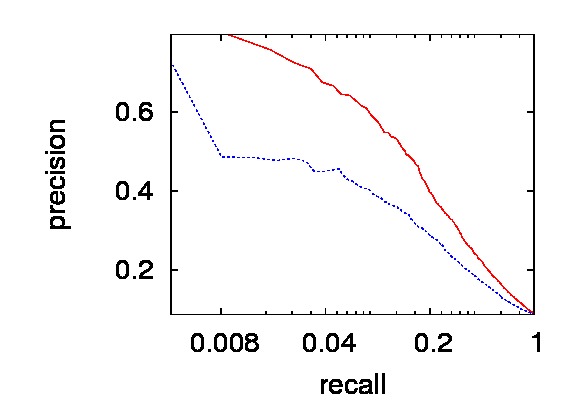

Supplement: Dataset S2 — Precision-recall figures for each tissue-specific network (red) versus the global (blue) network. (ZIP) [file pcbi.1002694.s002.zip › individual_figure/outer_nuclear_layer_retina_layer.txt.jpg]

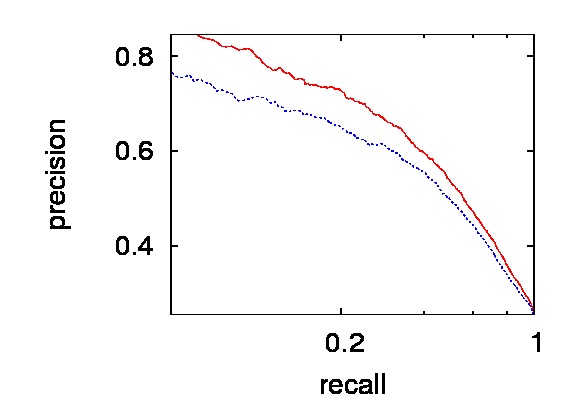

Supplement: Dataset S2 — Precision-recall figures for each tissue-specific network (red) versus the global (blue) network. (ZIP) [file pcbi.1002694.s002.zip › individual_figure/pyloric_antrum.txt.jpg]

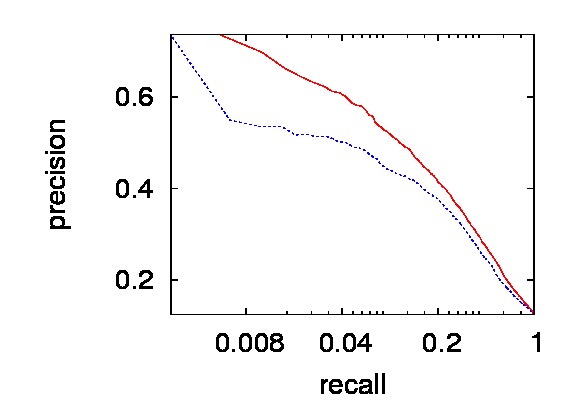

Supplement: Dataset S2 — Precision-recall figures for each tissue-specific network (red) versus the global (blue) network. (ZIP) [file pcbi.1002694.s002.zip › individual_figure/skeletal_muscle.txt.jpg]

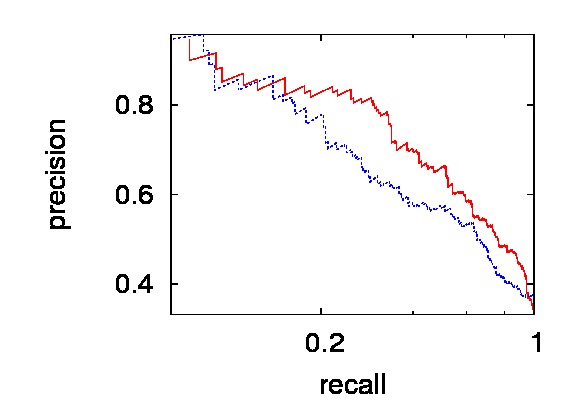

Supplement: Dataset S2 — Precision-recall figures for each tissue-specific network (red) versus the global (blue) network. (ZIP) [file pcbi.1002694.s002.zip › individual_figure/ventricular_zone_brain.txt.jpg]
