# Supplementary figures and images for: Tissue-Specific Functional Networks for Prioritizing Phenotype and Disease Genes (part 1 of 2)
Source: PLoS Comput Biol. 2012 Sep 27;8(9):e1002694. doi: 10.1371/journal.pcbi.1002694 (PMC3459891; doi:10.1371/journal.pcbi.1002694)

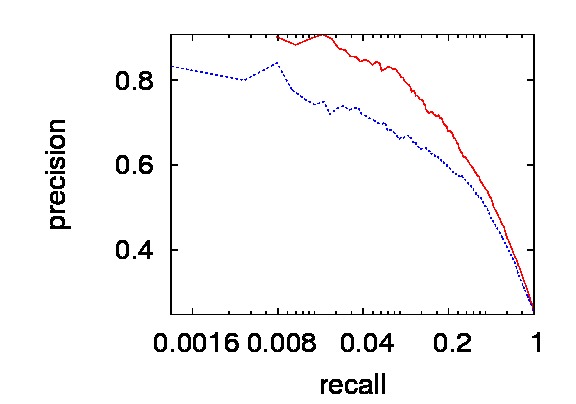

Supplement: Dataset S2 — Precision-recall figures for each tissue-specific network (red) versus the global (blue) network. (ZIP) [file pcbi.1002694.s002.zip › individual_figure/apex_of_caecum.txt.jpg]

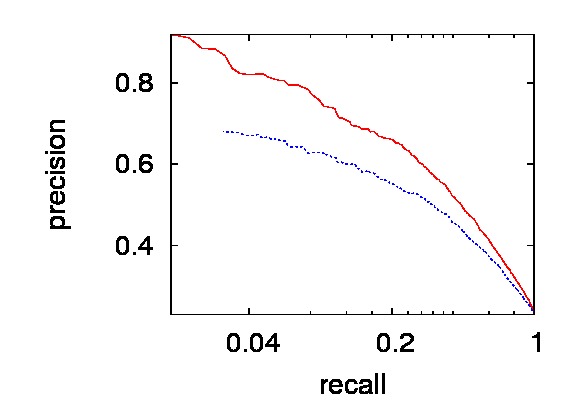

Supplement: Dataset S2 — Precision-recall figures for each tissue-specific network (red) versus the global (blue) network. (ZIP) [file pcbi.1002694.s002.zip › individual_figure/caecum.txt.jpg]

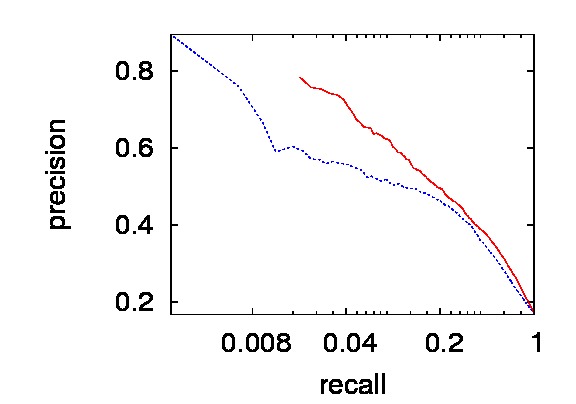

Supplement: Dataset S2 — Precision-recall figures for each tissue-specific network (red) versus the global (blue) network. (ZIP) [file pcbi.1002694.s002.zip › individual_figure/corpus_striatum.txt.jpg]

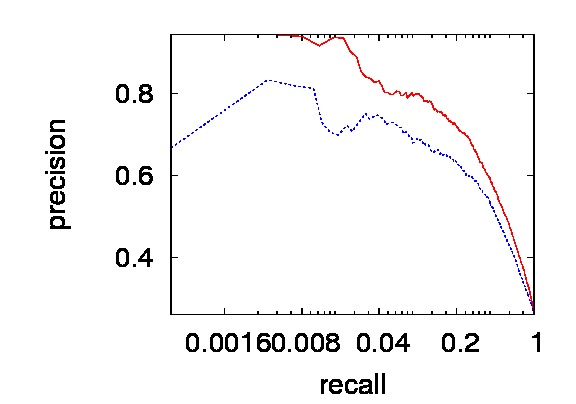

Supplement: Dataset S2 — Precision-recall figures for each tissue-specific network (red) versus the global (blue) network. (ZIP) [file pcbi.1002694.s002.zip › individual_figure/fundus_stomach.txt.jpg]

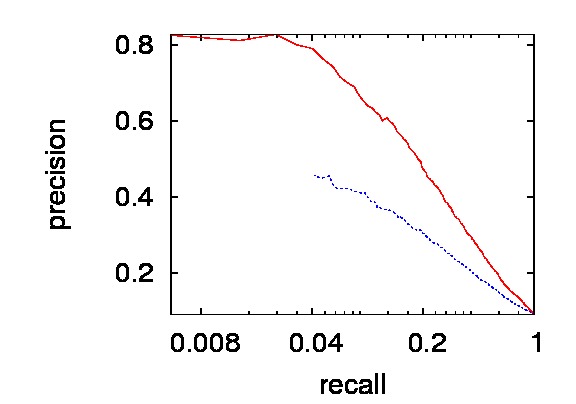

Supplement: Dataset S2 — Precision-recall figures for each tissue-specific network (red) versus the global (blue) network. (ZIP) [file pcbi.1002694.s002.zip › individual_figure/inner_nuclear_layer_retina_layer.txt.jpg]

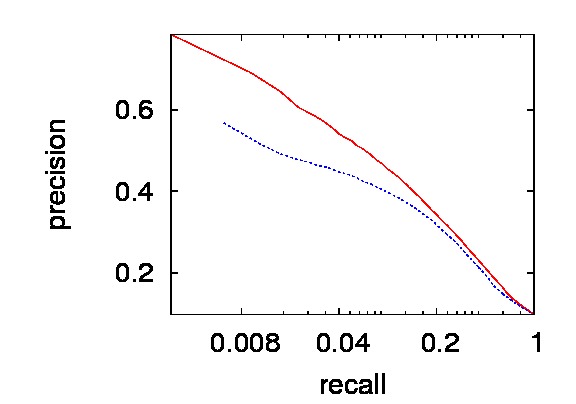

Supplement: Dataset S2 — Precision-recall figures for each tissue-specific network (red) versus the global (blue) network. (ZIP) [file pcbi.1002694.s002.zip › individual_figure/MA_0000006.txt.jpg]

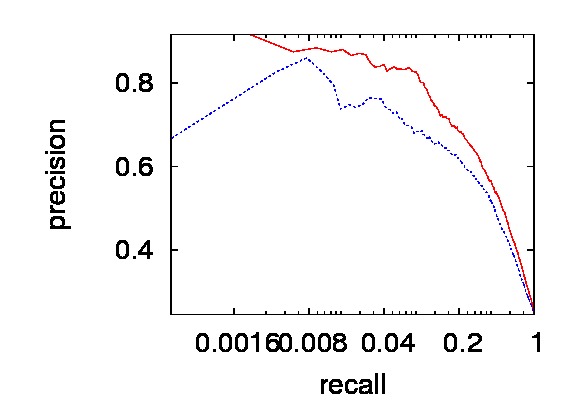

Supplement: Dataset S2 — Precision-recall figures for each tissue-specific network (red) versus the global (blue) network. (ZIP) [file pcbi.1002694.s002.zip › individual_figure/MA_0000009.txt.jpg]

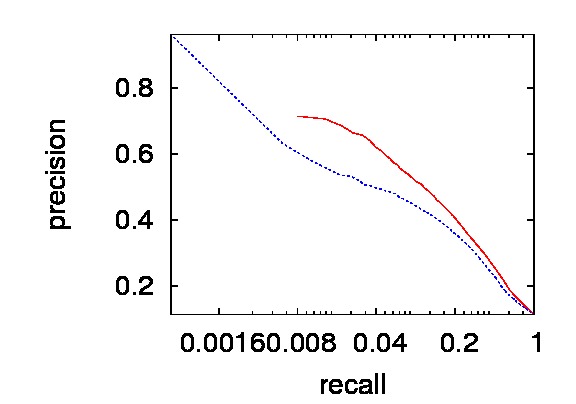

Supplement: Dataset S2 — Precision-recall figures for each tissue-specific network (red) versus the global (blue) network. (ZIP) [file pcbi.1002694.s002.zip › individual_figure/MA_0000010.txt.jpg]

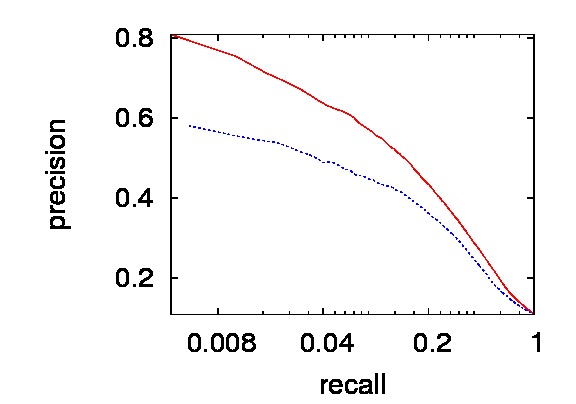

Supplement: Dataset S2 — Precision-recall figures for each tissue-specific network (red) versus the global (blue) network. (ZIP) [file pcbi.1002694.s002.zip › individual_figure/MA_0000013.txt.jpg]

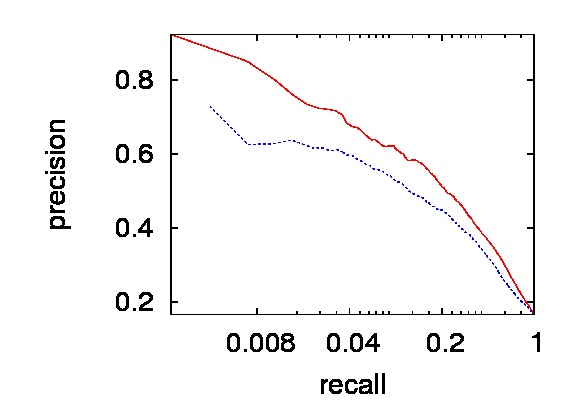

Supplement: Dataset S2 — Precision-recall figures for each tissue-specific network (red) versus the global (blue) network. (ZIP) [file pcbi.1002694.s002.zip › individual_figure/MA_0000014.txt.jpg]

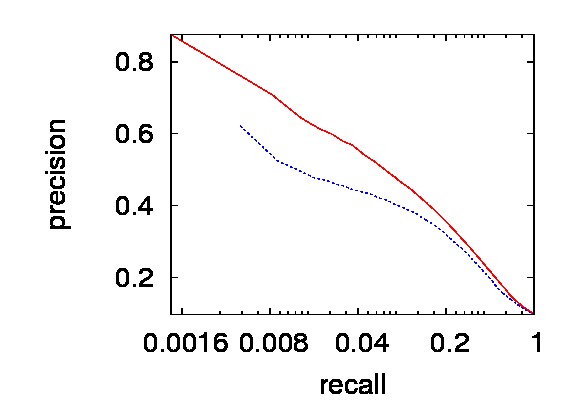

Supplement: Dataset S2 — Precision-recall figures for each tissue-specific network (red) versus the global (blue) network. (ZIP) [file pcbi.1002694.s002.zip › individual_figure/MA_0000016.txt.jpg]

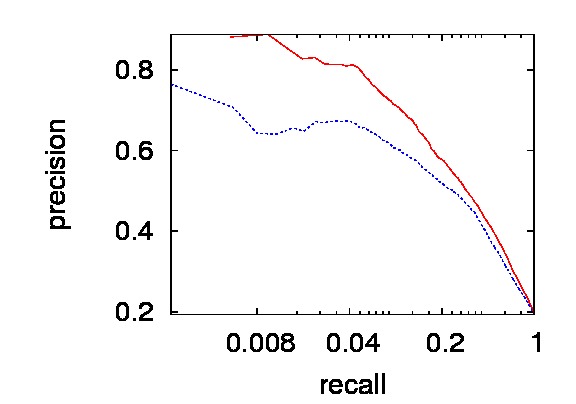

Supplement: Dataset S2 — Precision-recall figures for each tissue-specific network (red) versus the global (blue) network. (ZIP) [file pcbi.1002694.s002.zip › individual_figure/MA_0000018.txt.jpg]

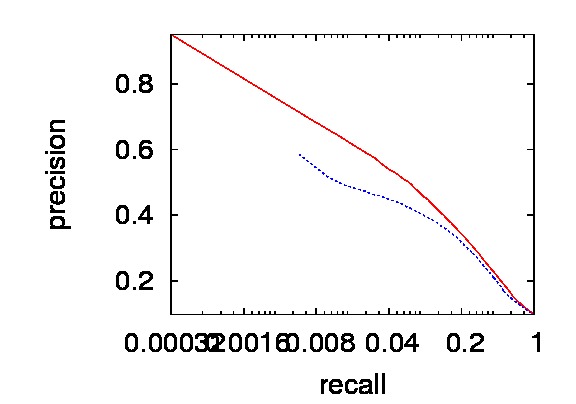

Supplement: Dataset S2 — Precision-recall figures for each tissue-specific network (red) versus the global (blue) network. (ZIP) [file pcbi.1002694.s002.zip › individual_figure/MA_0000023.txt.jpg]

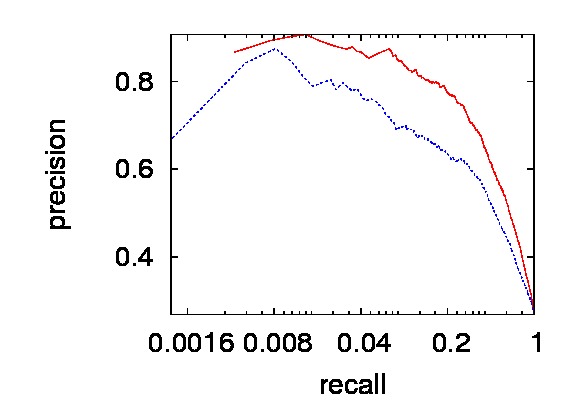

Supplement: Dataset S2 — Precision-recall figures for each tissue-specific network (red) versus the global (blue) network. (ZIP) [file pcbi.1002694.s002.zip › individual_figure/MA_0000025.txt.jpg]

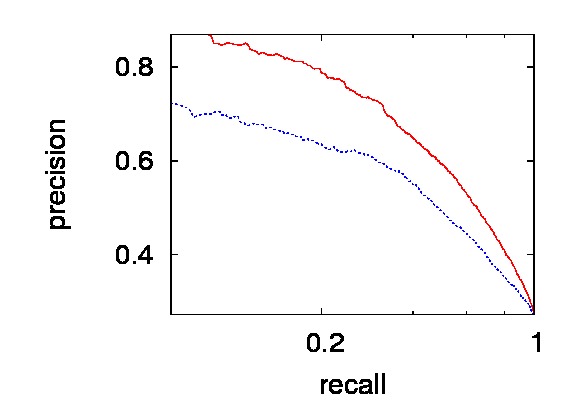

Supplement: Dataset S2 — Precision-recall figures for each tissue-specific network (red) versus the global (blue) network. (ZIP) [file pcbi.1002694.s002.zip › individual_figure/MA_0000037.txt.jpg]

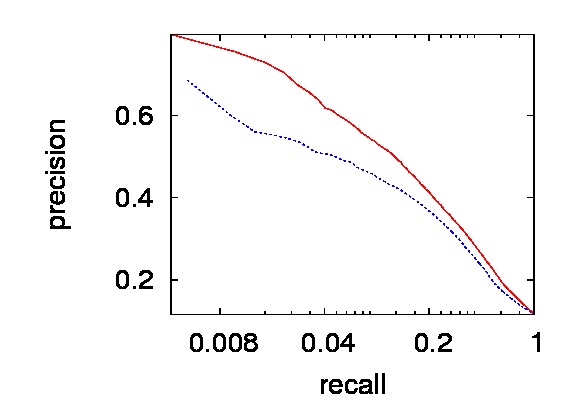

Supplement: Dataset S2 — Precision-recall figures for each tissue-specific network (red) versus the global (blue) network. (ZIP) [file pcbi.1002694.s002.zip › individual_figure/MA_0000072.txt.jpg]

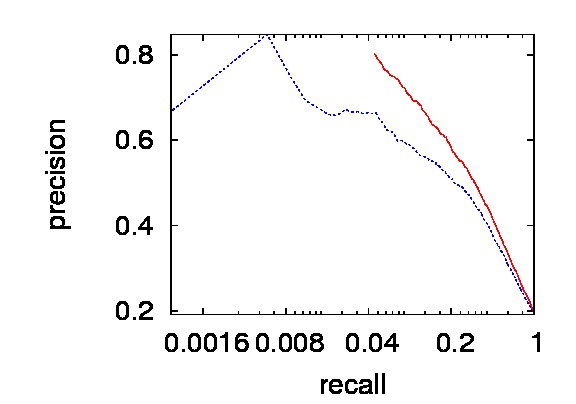

Supplement: Dataset S2 — Precision-recall figures for each tissue-specific network (red) versus the global (blue) network. (ZIP) [file pcbi.1002694.s002.zip › individual_figure/MA_0000116.txt.jpg]

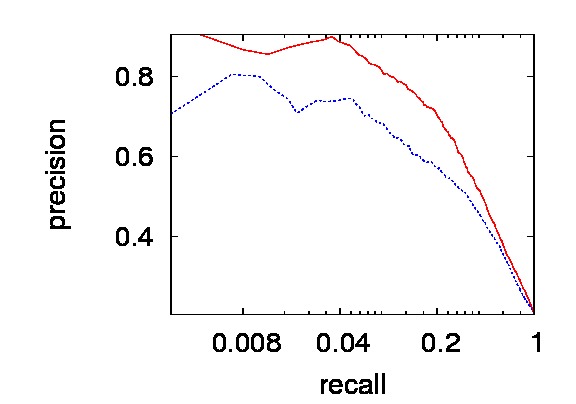

Supplement: Dataset S2 — Precision-recall figures for each tissue-specific network (red) versus the global (blue) network. (ZIP) [file pcbi.1002694.s002.zip › individual_figure/MA_0000120.txt.jpg]

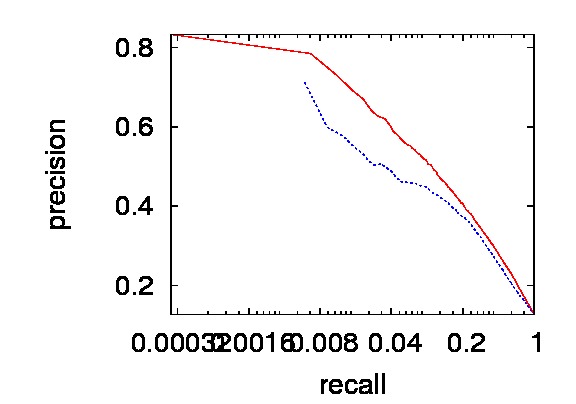

Supplement: Dataset S2 — Precision-recall figures for each tissue-specific network (red) versus the global (blue) network. (ZIP) [file pcbi.1002694.s002.zip › individual_figure/MA_0000133.txt.jpg]

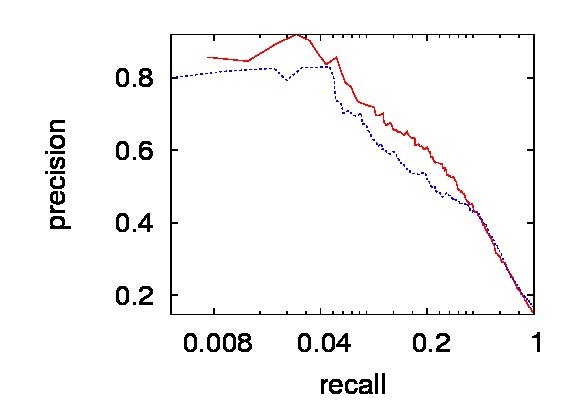

Supplement: Dataset S2 — Precision-recall figures for each tissue-specific network (red) versus the global (blue) network. (ZIP) [file pcbi.1002694.s002.zip › individual_figure/MA_0000134.txt.jpg]

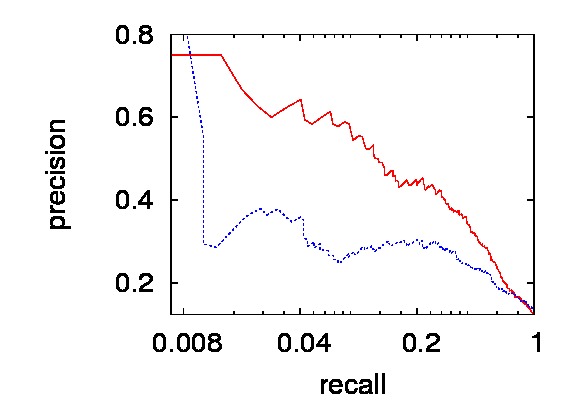

Supplement: Dataset S2 — Precision-recall figures for each tissue-specific network (red) versus the global (blue) network. (ZIP) [file pcbi.1002694.s002.zip › individual_figure/MA_0000139.txt.jpg]

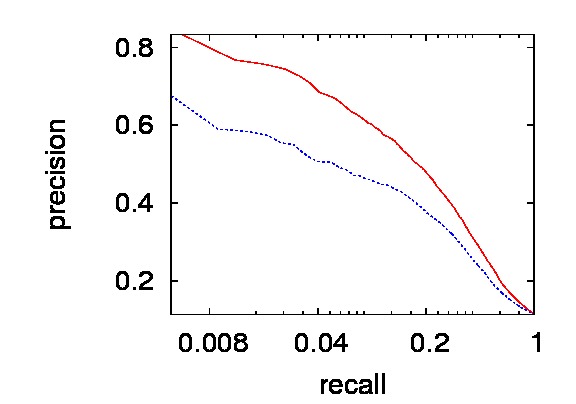

Supplement: Dataset S2 — Precision-recall figures for each tissue-specific network (red) versus the global (blue) network. (ZIP) [file pcbi.1002694.s002.zip › individual_figure/MA_0000141.txt.jpg]

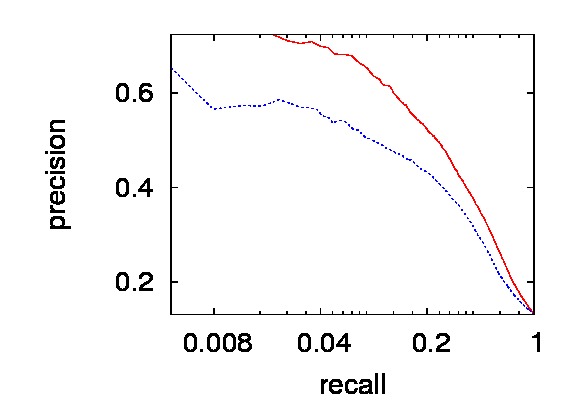

Supplement: Dataset S2 — Precision-recall figures for each tissue-specific network (red) versus the global (blue) network. (ZIP) [file pcbi.1002694.s002.zip › individual_figure/MA_0000142.txt.jpg]

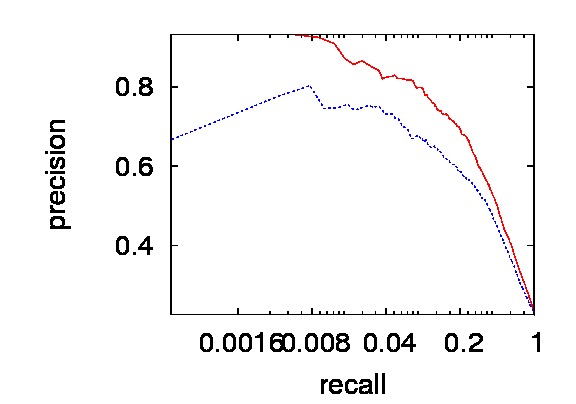

Supplement: Dataset S2 — Precision-recall figures for each tissue-specific network (red) versus the global (blue) network. (ZIP) [file pcbi.1002694.s002.zip › individual_figure/MA_0000145.txt.jpg]

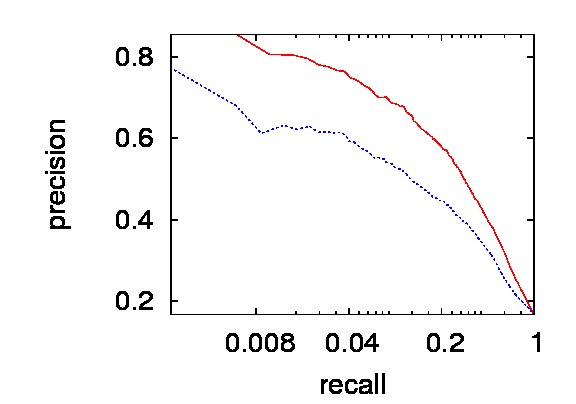

Supplement: Dataset S2 — Precision-recall figures for each tissue-specific network (red) versus the global (blue) network. (ZIP) [file pcbi.1002694.s002.zip › individual_figure/MA_0000151.txt.jpg]

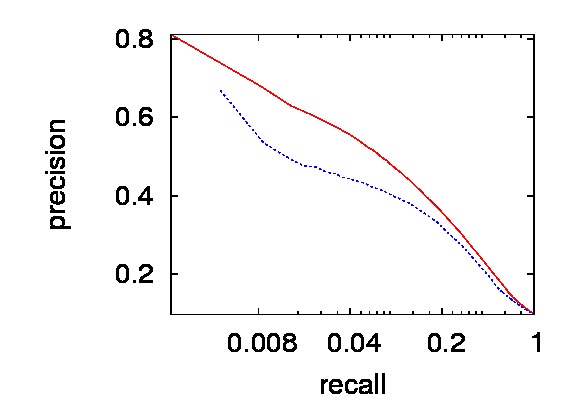

Supplement: Dataset S2 — Precision-recall figures for each tissue-specific network (red) versus the global (blue) network. (ZIP) [file pcbi.1002694.s002.zip › individual_figure/MA_0000167.txt.jpg]

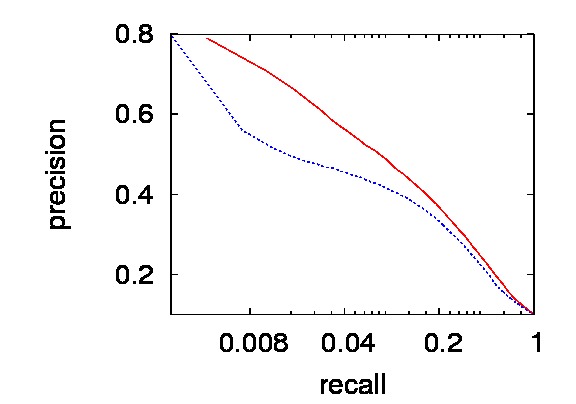

Supplement: Dataset S2 — Precision-recall figures for each tissue-specific network (red) versus the global (blue) network. (ZIP) [file pcbi.1002694.s002.zip › individual_figure/MA_0000168.txt.jpg]

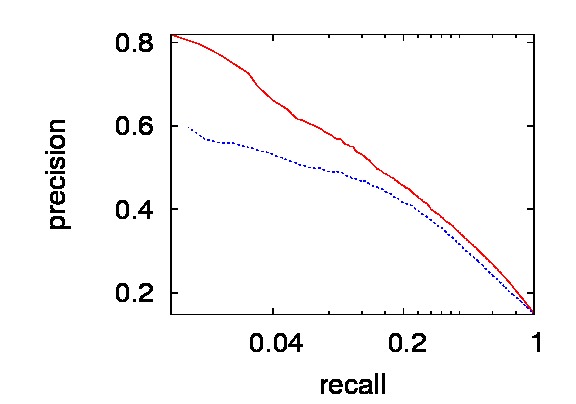

Supplement: Dataset S2 — Precision-recall figures for each tissue-specific network (red) versus the global (blue) network. (ZIP) [file pcbi.1002694.s002.zip › individual_figure/MA_0000169.txt.jpg]

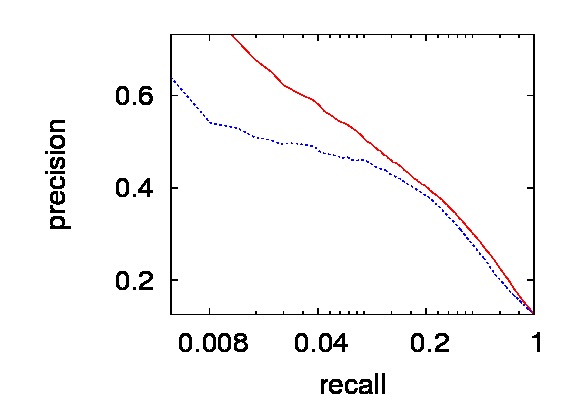

Supplement: Dataset S2 — Precision-recall figures for each tissue-specific network (red) versus the global (blue) network. (ZIP) [file pcbi.1002694.s002.zip › individual_figure/MA_0000170.txt.jpg]

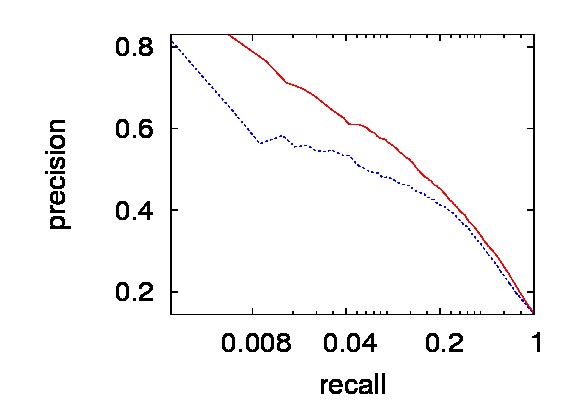

Supplement: Dataset S2 — Precision-recall figures for each tissue-specific network (red) versus the global (blue) network. (ZIP) [file pcbi.1002694.s002.zip › individual_figure/MA_0000171.txt.jpg]

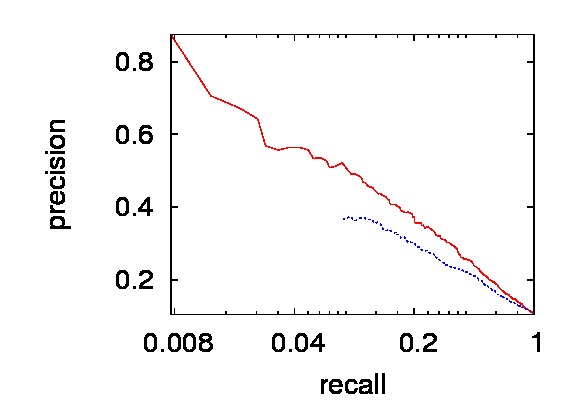

Supplement: Dataset S2 — Precision-recall figures for each tissue-specific network (red) versus the global (blue) network. (ZIP) [file pcbi.1002694.s002.zip › individual_figure/MA_0000173.txt.jpg]

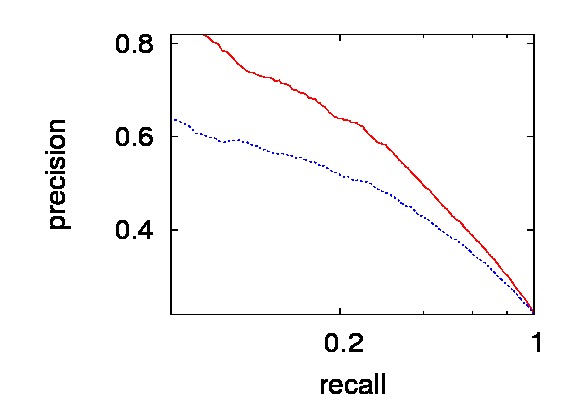

Supplement: Dataset S2 — Precision-recall figures for each tissue-specific network (red) versus the global (blue) network. (ZIP) [file pcbi.1002694.s002.zip › individual_figure/MA_0000176.txt.jpg]

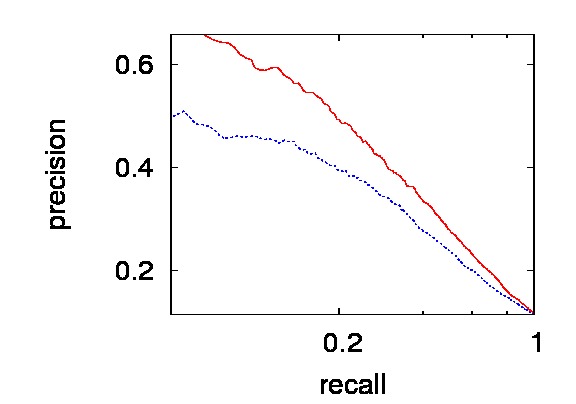

Supplement: Dataset S2 — Precision-recall figures for each tissue-specific network (red) versus the global (blue) network. (ZIP) [file pcbi.1002694.s002.zip › individual_figure/MA_0000179.txt.jpg]

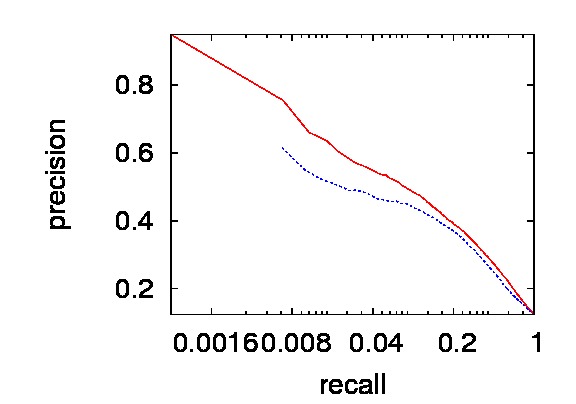

Supplement: Dataset S2 — Precision-recall figures for each tissue-specific network (red) versus the global (blue) network. (ZIP) [file pcbi.1002694.s002.zip › individual_figure/MA_0000183.txt.jpg]

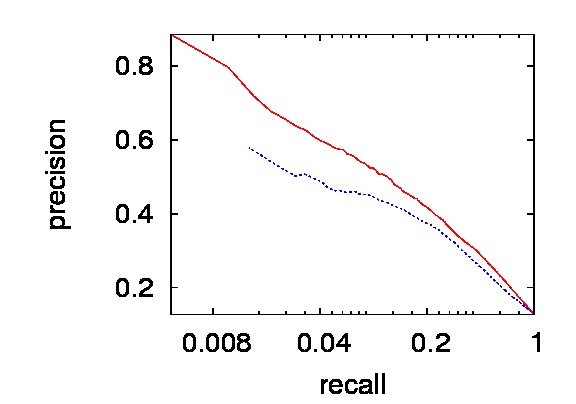

Supplement: Dataset S2 — Precision-recall figures for each tissue-specific network (red) versus the global (blue) network. (ZIP) [file pcbi.1002694.s002.zip › individual_figure/MA_0000185.txt.jpg]

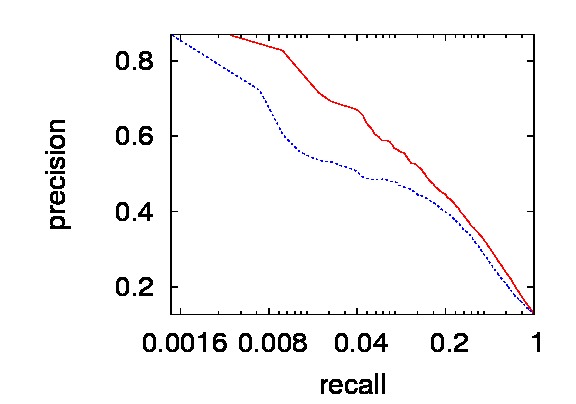

Supplement: Dataset S2 — Precision-recall figures for each tissue-specific network (red) versus the global (blue) network. (ZIP) [file pcbi.1002694.s002.zip › individual_figure/MA_0000189.txt.jpg]

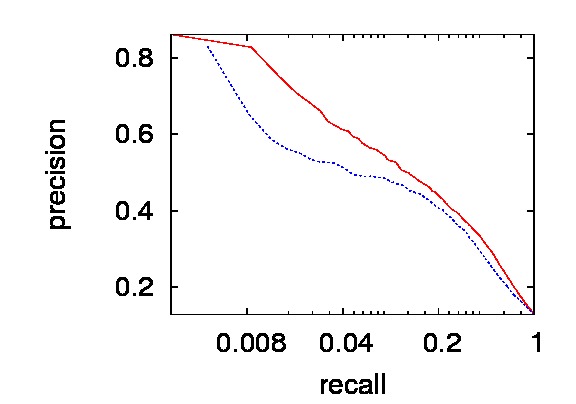

Supplement: Dataset S2 — Precision-recall figures for each tissue-specific network (red) versus the global (blue) network. (ZIP) [file pcbi.1002694.s002.zip › individual_figure/MA_0000191.txt.jpg]

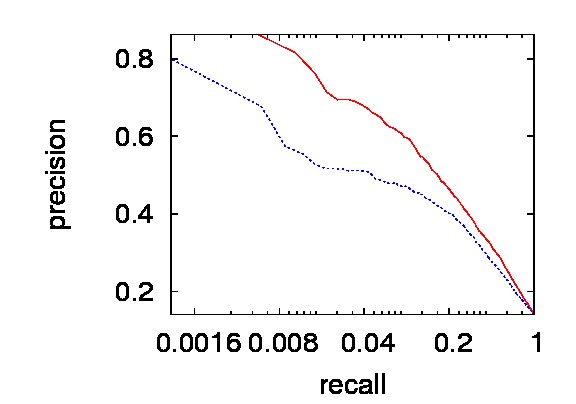

Supplement: Dataset S2 — Precision-recall figures for each tissue-specific network (red) versus the global (blue) network. (ZIP) [file pcbi.1002694.s002.zip › individual_figure/MA_0000194.txt.jpg]

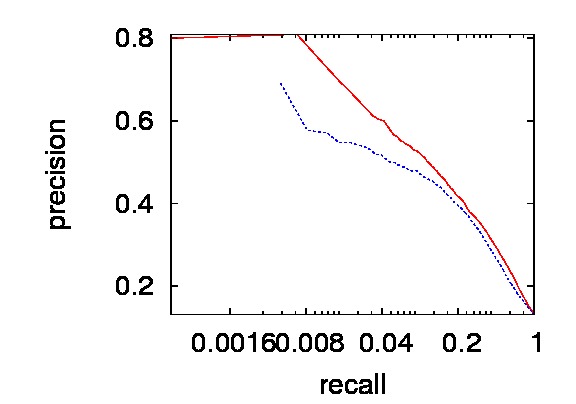

Supplement: Dataset S2 — Precision-recall figures for each tissue-specific network (red) versus the global (blue) network. (ZIP) [file pcbi.1002694.s002.zip › individual_figure/MA_0000195.txt.jpg]

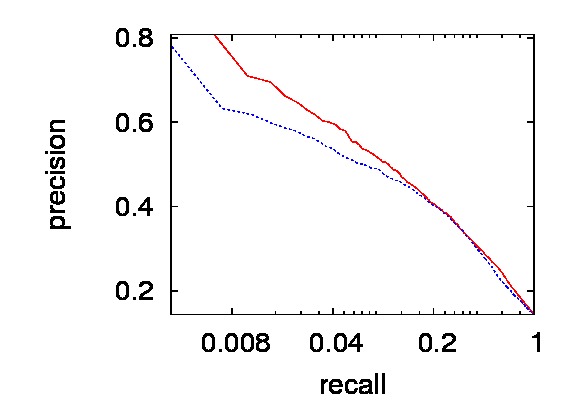

Supplement: Dataset S2 — Precision-recall figures for each tissue-specific network (red) versus the global (blue) network. (ZIP) [file pcbi.1002694.s002.zip › individual_figure/MA_0000197.txt.jpg]

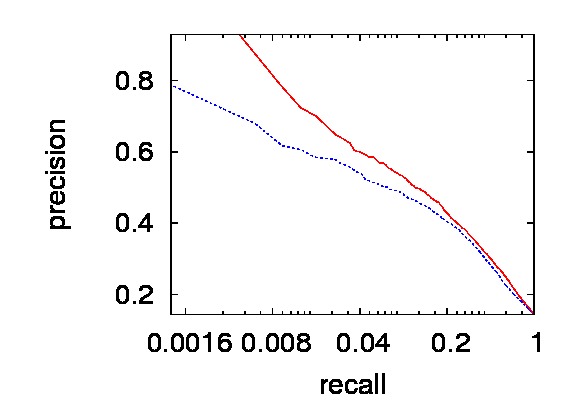

Supplement: Dataset S2 — Precision-recall figures for each tissue-specific network (red) versus the global (blue) network. (ZIP) [file pcbi.1002694.s002.zip › individual_figure/MA_0000198.txt.jpg]

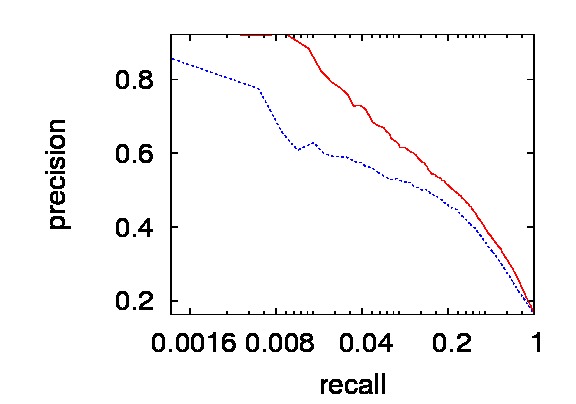

Supplement: Dataset S2 — Precision-recall figures for each tissue-specific network (red) versus the global (blue) network. (ZIP) [file pcbi.1002694.s002.zip › individual_figure/MA_0000207.txt.jpg]

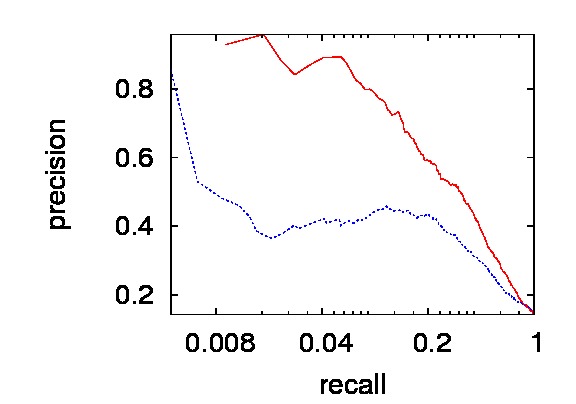

Supplement: Dataset S2 — Precision-recall figures for each tissue-specific network (red) versus the global (blue) network. (ZIP) [file pcbi.1002694.s002.zip › individual_figure/MA_0000214.txt.jpg]

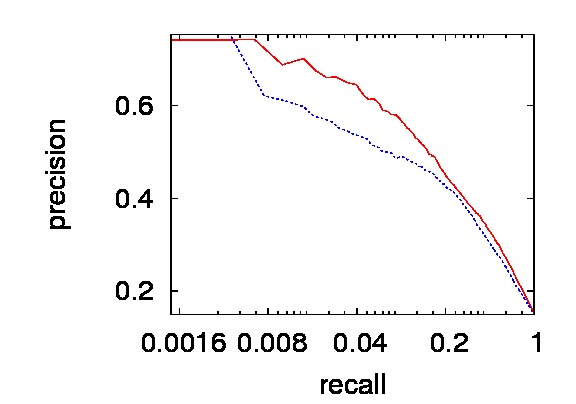

Supplement: Dataset S2 — Precision-recall figures for each tissue-specific network (red) versus the global (blue) network. (ZIP) [file pcbi.1002694.s002.zip › individual_figure/MA_0000216.txt.jpg]

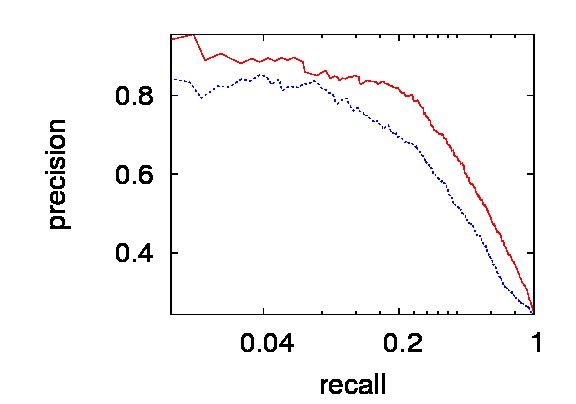

Supplement: Dataset S2 — Precision-recall figures for each tissue-specific network (red) versus the global (blue) network. (ZIP) [file pcbi.1002694.s002.zip › individual_figure/MA_0000232.txt.jpg]

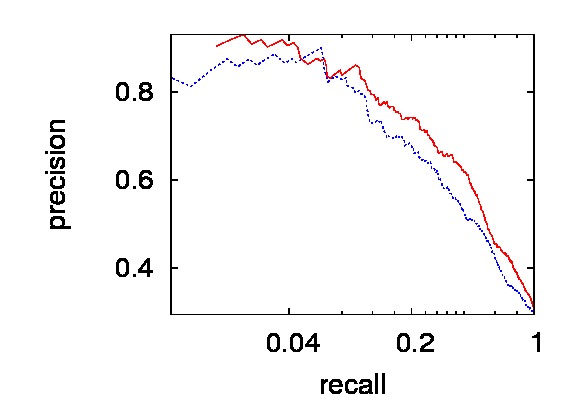

Supplement: Dataset S2 — Precision-recall figures for each tissue-specific network (red) versus the global (blue) network. (ZIP) [file pcbi.1002694.s002.zip › individual_figure/MA_0000236.txt.jpg]

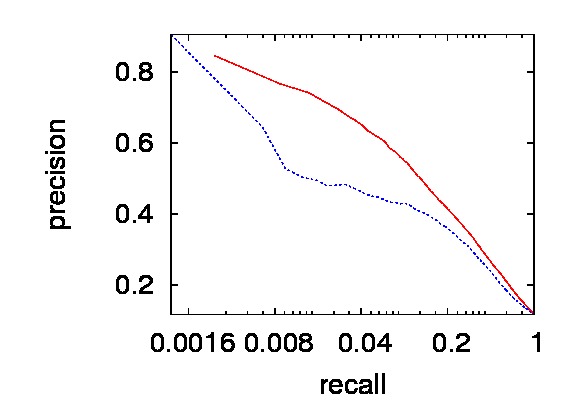

Supplement: Dataset S2 — Precision-recall figures for each tissue-specific network (red) versus the global (blue) network. (ZIP) [file pcbi.1002694.s002.zip › individual_figure/MA_0000261.txt.jpg]

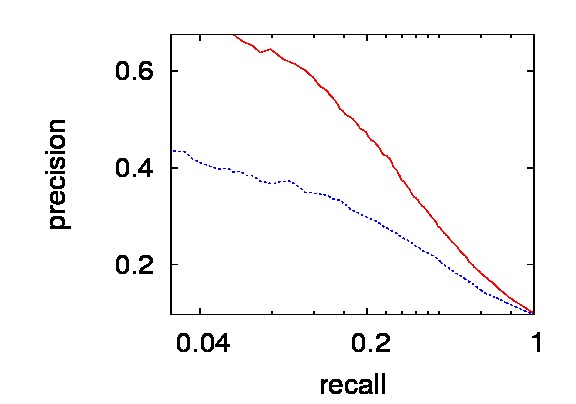

Supplement: Dataset S2 — Precision-recall figures for each tissue-specific network (red) versus the global (blue) network. (ZIP) [file pcbi.1002694.s002.zip › individual_figure/MA_0000276.txt.jpg]

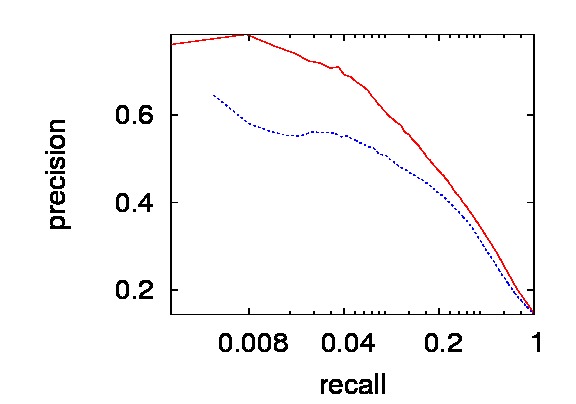

Supplement: Dataset S2 — Precision-recall figures for each tissue-specific network (red) versus the global (blue) network. (ZIP) [file pcbi.1002694.s002.zip › individual_figure/MA_0000323.txt.jpg]

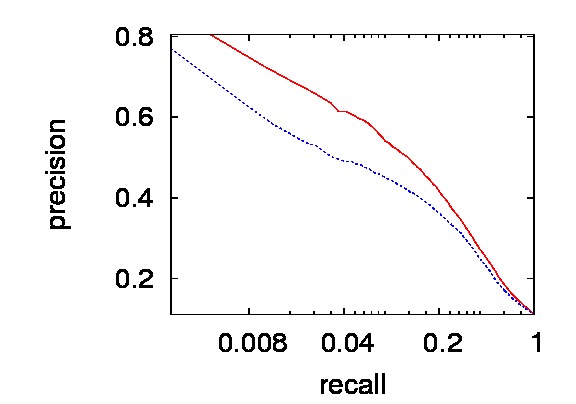

Supplement: Dataset S2 — Precision-recall figures for each tissue-specific network (red) versus the global (blue) network. (ZIP) [file pcbi.1002694.s002.zip › individual_figure/MA_0000324.txt.jpg]

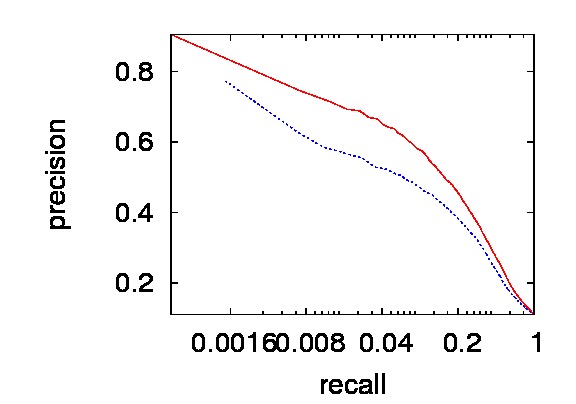

Supplement: Dataset S2 — Precision-recall figures for each tissue-specific network (red) versus the global (blue) network. (ZIP) [file pcbi.1002694.s002.zip › individual_figure/MA_0000325.txt.jpg]

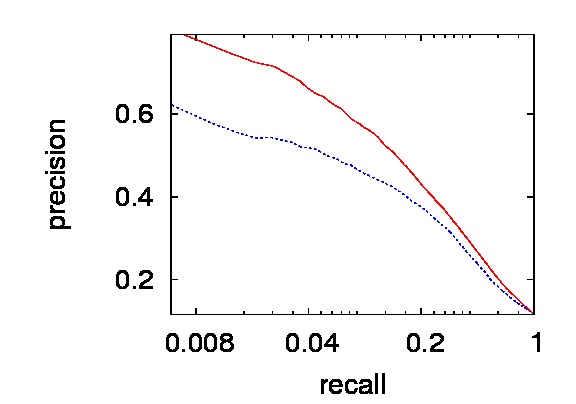

Supplement: Dataset S2 — Precision-recall figures for each tissue-specific network (red) versus the global (blue) network. (ZIP) [file pcbi.1002694.s002.zip › individual_figure/MA_0000327.txt.jpg]

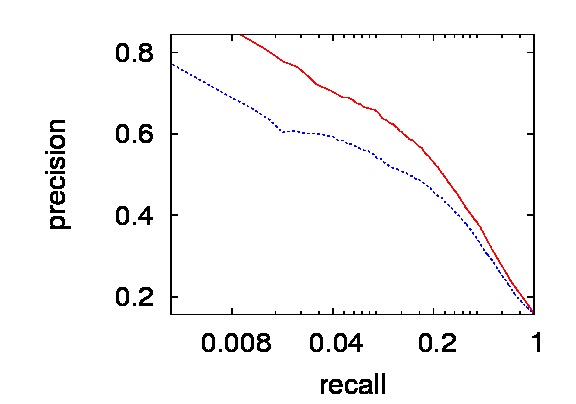

Supplement: Dataset S2 — Precision-recall figures for each tissue-specific network (red) versus the global (blue) network. (ZIP) [file pcbi.1002694.s002.zip › individual_figure/MA_0000328.txt.jpg]

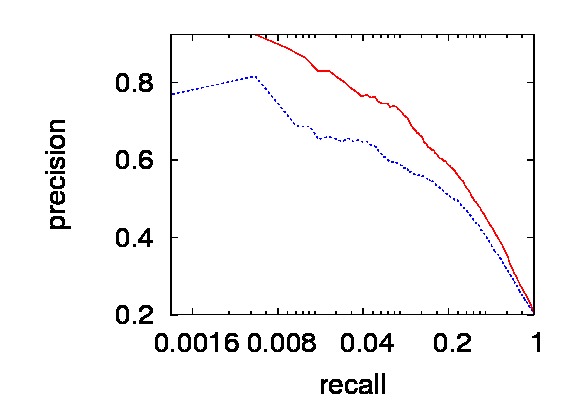

Supplement: Dataset S2 — Precision-recall figures for each tissue-specific network (red) versus the global (blue) network. (ZIP) [file pcbi.1002694.s002.zip › individual_figure/MA_0000333.txt.jpg]

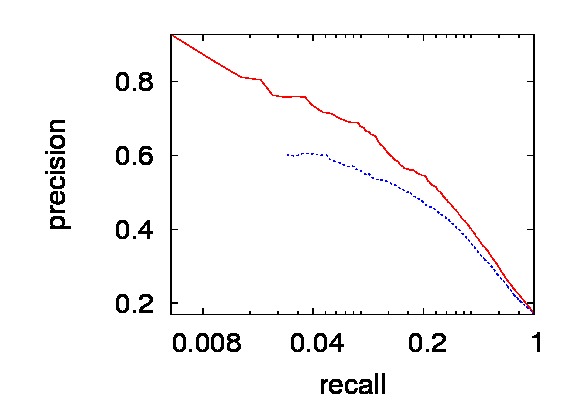

Supplement: Dataset S2 — Precision-recall figures for each tissue-specific network (red) versus the global (blue) network. (ZIP) [file pcbi.1002694.s002.zip › individual_figure/MA_0000337.txt.jpg]

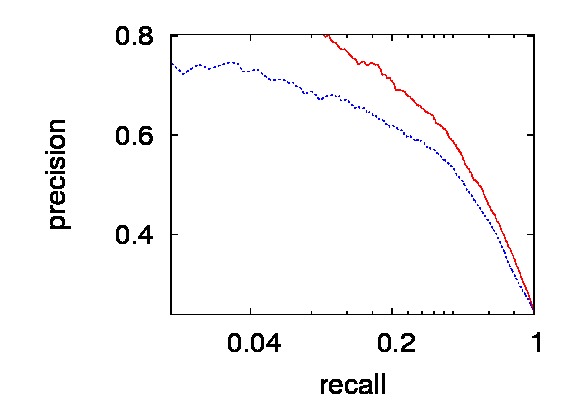

Supplement: Dataset S2 — Precision-recall figures for each tissue-specific network (red) versus the global (blue) network. (ZIP) [file pcbi.1002694.s002.zip › individual_figure/MA_0000338.txt.jpg]

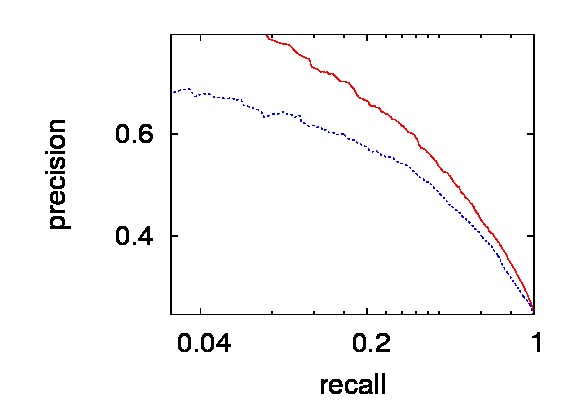

Supplement: Dataset S2 — Precision-recall figures for each tissue-specific network (red) versus the global (blue) network. (ZIP) [file pcbi.1002694.s002.zip › individual_figure/MA_0000339.txt.jpg]

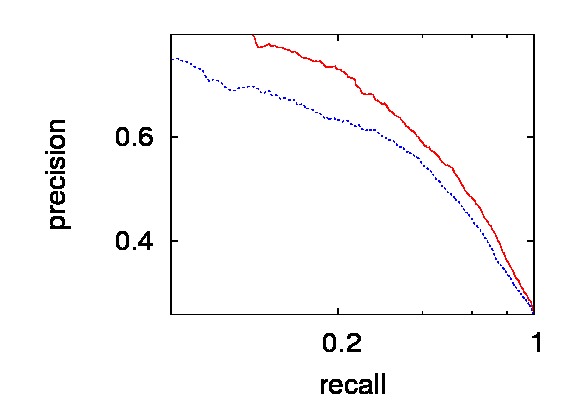

Supplement: Dataset S2 — Precision-recall figures for each tissue-specific network (red) versus the global (blue) network. (ZIP) [file pcbi.1002694.s002.zip › individual_figure/MA_0000340.txt.jpg]

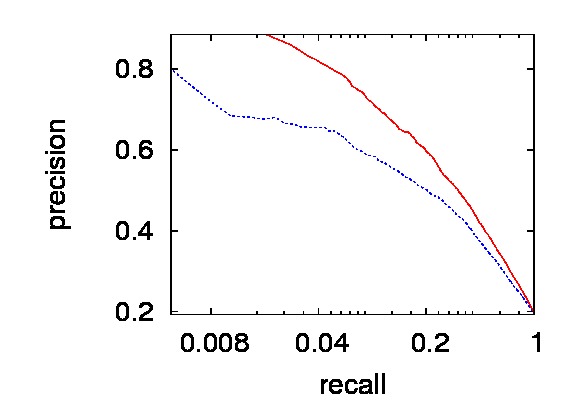

Supplement: Dataset S2 — Precision-recall figures for each tissue-specific network (red) versus the global (blue) network. (ZIP) [file pcbi.1002694.s002.zip › individual_figure/MA_0000341.txt.jpg]

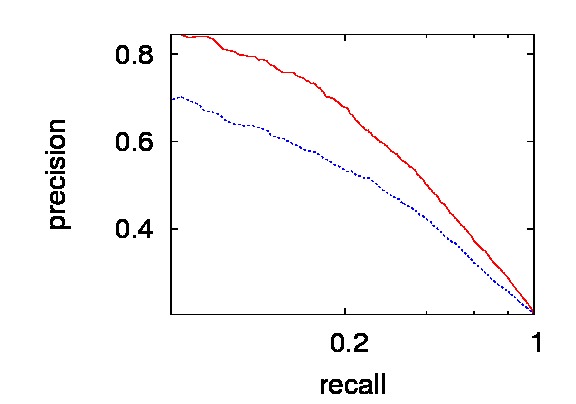

Supplement: Dataset S2 — Precision-recall figures for each tissue-specific network (red) versus the global (blue) network. (ZIP) [file pcbi.1002694.s002.zip › individual_figure/MA_0000346.txt.jpg]

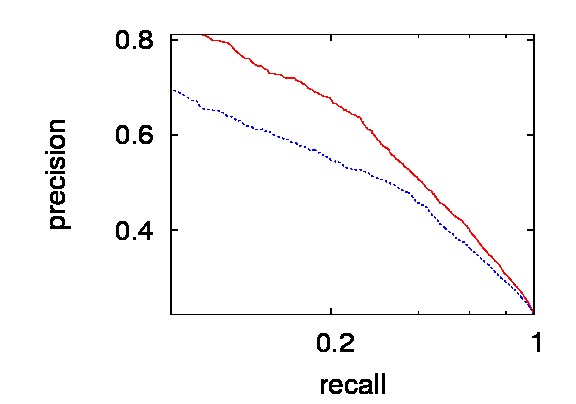

Supplement: Dataset S2 — Precision-recall figures for each tissue-specific network (red) versus the global (blue) network. (ZIP) [file pcbi.1002694.s002.zip › individual_figure/MA_0000347.txt.jpg]

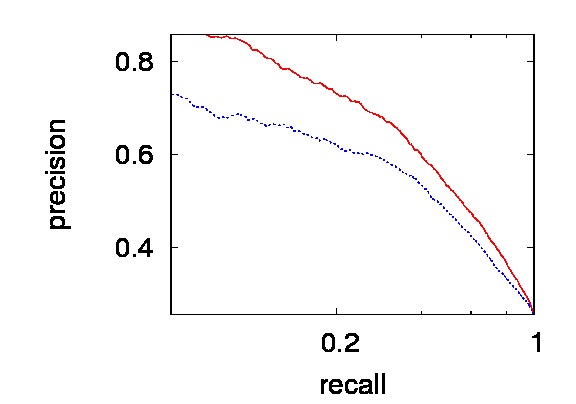

Supplement: Dataset S2 — Precision-recall figures for each tissue-specific network (red) versus the global (blue) network. (ZIP) [file pcbi.1002694.s002.zip › individual_figure/MA_0000349.txt.jpg]

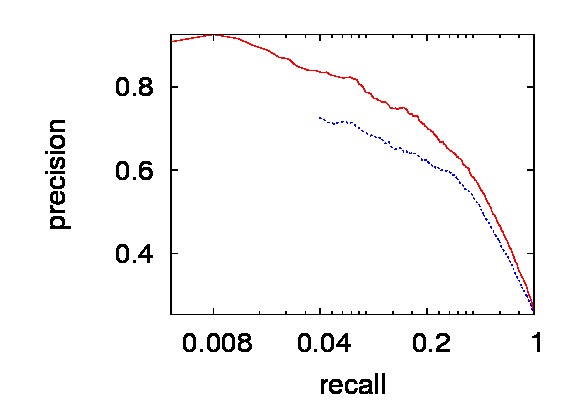

Supplement: Dataset S2 — Precision-recall figures for each tissue-specific network (red) versus the global (blue) network. (ZIP) [file pcbi.1002694.s002.zip › individual_figure/MA_0000352.txt.jpg]

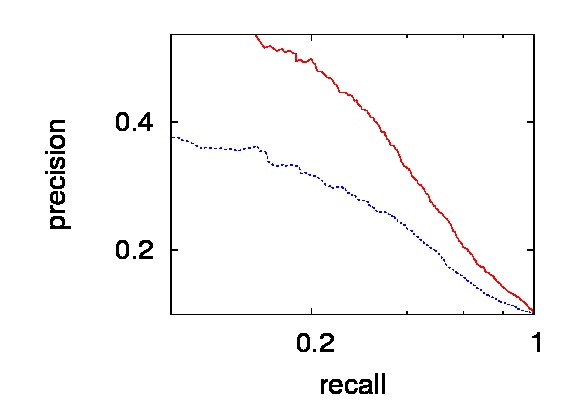

Supplement: Dataset S2 — Precision-recall figures for each tissue-specific network (red) versus the global (blue) network. (ZIP) [file pcbi.1002694.s002.zip › individual_figure/MA_0000353.txt.jpg]

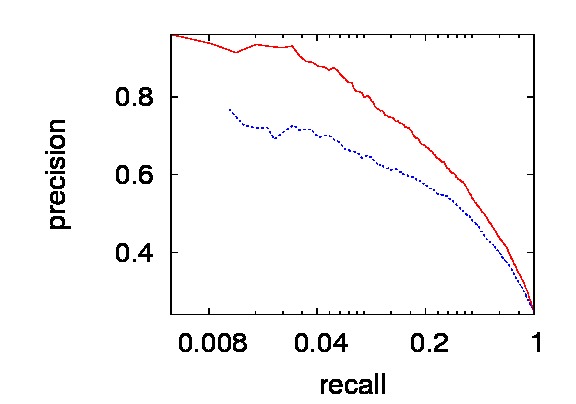

Supplement: Dataset S2 — Precision-recall figures for each tissue-specific network (red) versus the global (blue) network. (ZIP) [file pcbi.1002694.s002.zip › individual_figure/MA_0000356.txt.jpg]

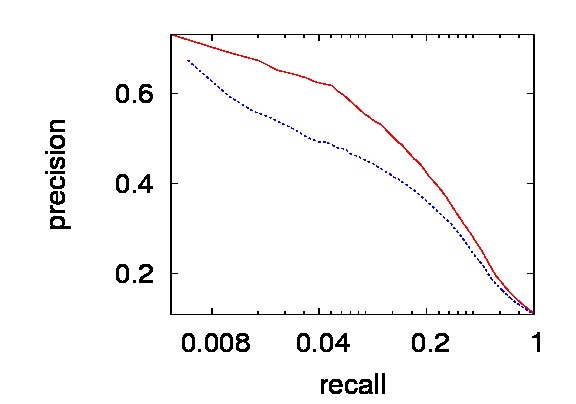

Supplement: Dataset S2 — Precision-recall figures for each tissue-specific network (red) versus the global (blue) network. (ZIP) [file pcbi.1002694.s002.zip › individual_figure/MA_0000358.txt.jpg]

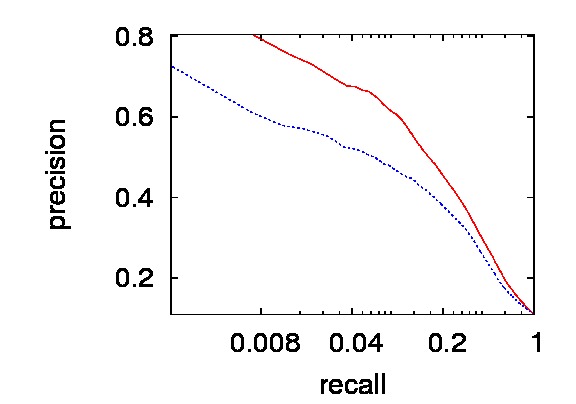

Supplement: Dataset S2 — Precision-recall figures for each tissue-specific network (red) versus the global (blue) network. (ZIP) [file pcbi.1002694.s002.zip › individual_figure/MA_0000368.txt.jpg]

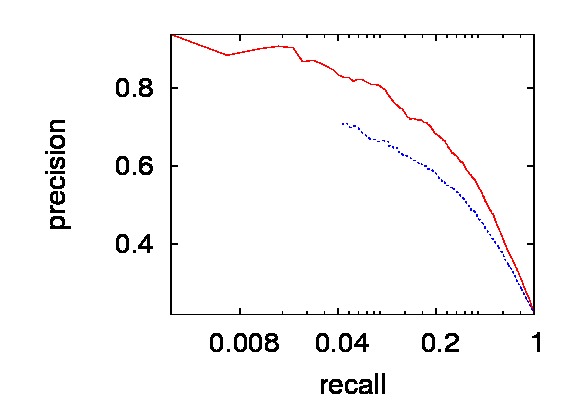

Supplement: Dataset S2 — Precision-recall figures for each tissue-specific network (red) versus the global (blue) network. (ZIP) [file pcbi.1002694.s002.zip › individual_figure/MA_0000380.txt.jpg]

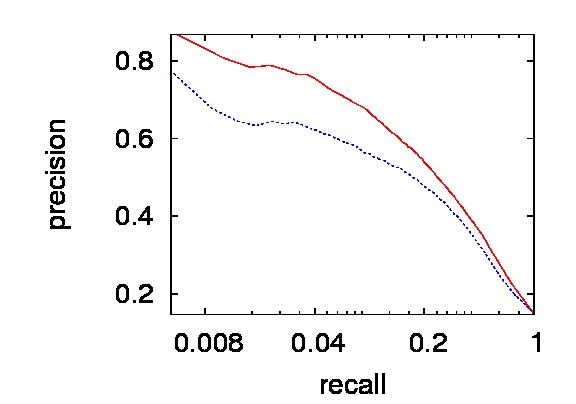

Supplement: Dataset S2 — Precision-recall figures for each tissue-specific network (red) versus the global (blue) network. (ZIP) [file pcbi.1002694.s002.zip › individual_figure/MA_0000381.txt.jpg]

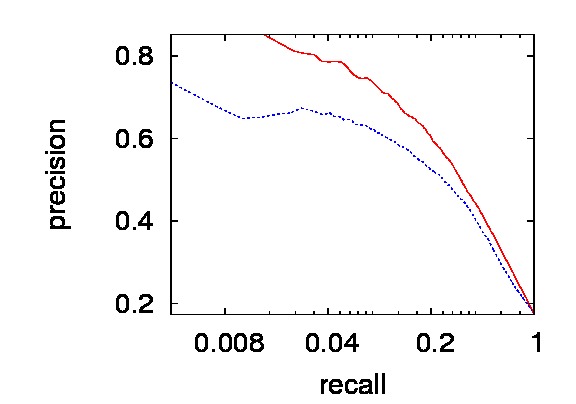

Supplement: Dataset S2 — Precision-recall figures for each tissue-specific network (red) versus the global (blue) network. (ZIP) [file pcbi.1002694.s002.zip › individual_figure/MA_0000384.txt.jpg]

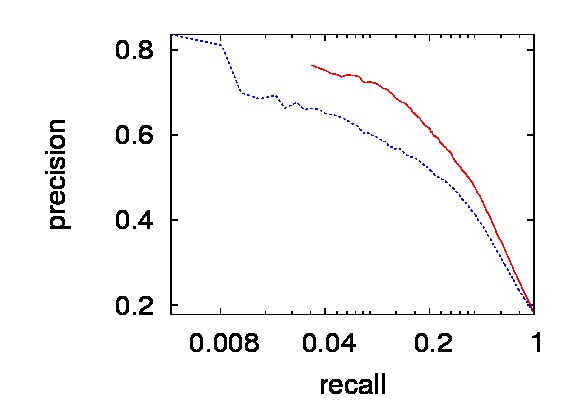

Supplement: Dataset S2 — Precision-recall figures for each tissue-specific network (red) versus the global (blue) network. (ZIP) [file pcbi.1002694.s002.zip › individual_figure/MA_0000386.txt.jpg]

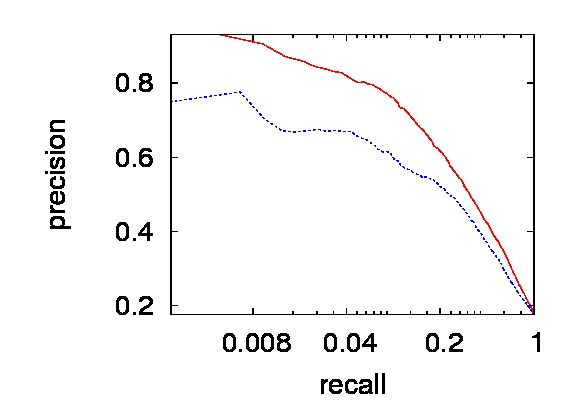

Supplement: Dataset S2 — Precision-recall figures for each tissue-specific network (red) versus the global (blue) network. (ZIP) [file pcbi.1002694.s002.zip › individual_figure/MA_0000389.txt.jpg]

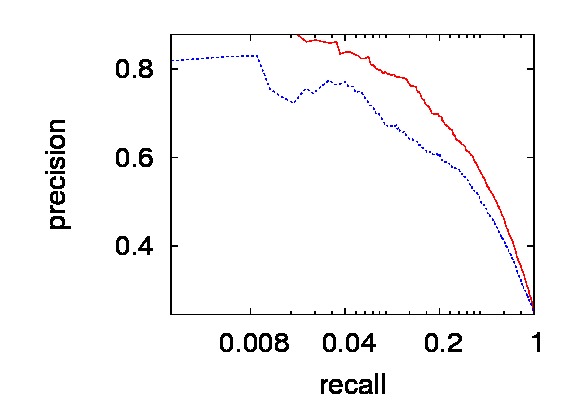

Supplement: Dataset S2 — Precision-recall figures for each tissue-specific network (red) versus the global (blue) network. (ZIP) [file pcbi.1002694.s002.zip › individual_figure/MA_0000392.txt.jpg]

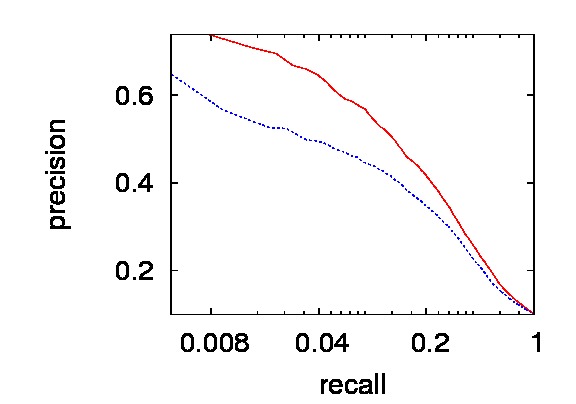

Supplement: Dataset S2 — Precision-recall figures for each tissue-specific network (red) versus the global (blue) network. (ZIP) [file pcbi.1002694.s002.zip › individual_figure/MA_0000396.txt.jpg]

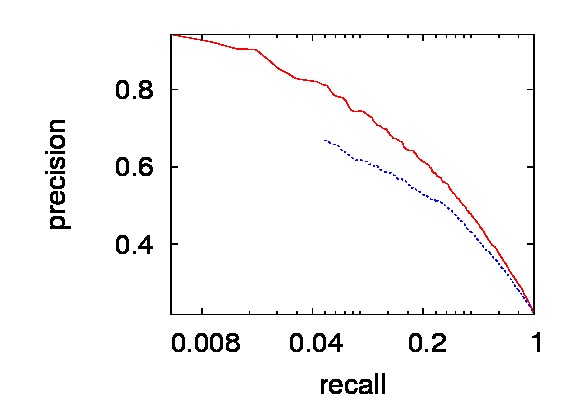

Supplement: Dataset S2 — Precision-recall figures for each tissue-specific network (red) versus the global (blue) network. (ZIP) [file pcbi.1002694.s002.zip › individual_figure/MA_0000397.txt.jpg]

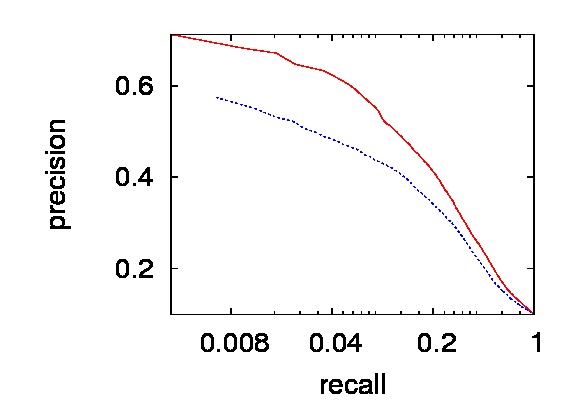

Supplement: Dataset S2 — Precision-recall figures for each tissue-specific network (red) versus the global (blue) network. (ZIP) [file pcbi.1002694.s002.zip › individual_figure/MA_0000411.txt.jpg]

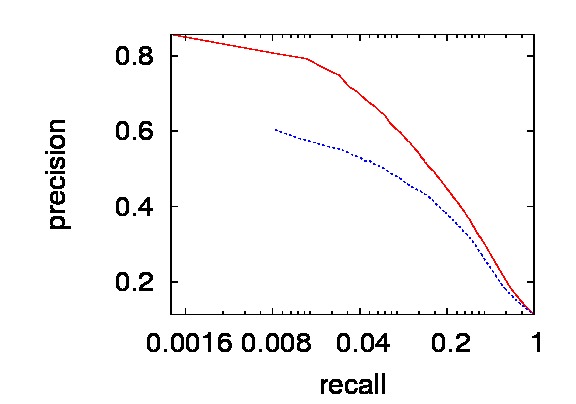

Supplement: Dataset S2 — Precision-recall figures for each tissue-specific network (red) versus the global (blue) network. (ZIP) [file pcbi.1002694.s002.zip › individual_figure/MA_0000415.txt.jpg]

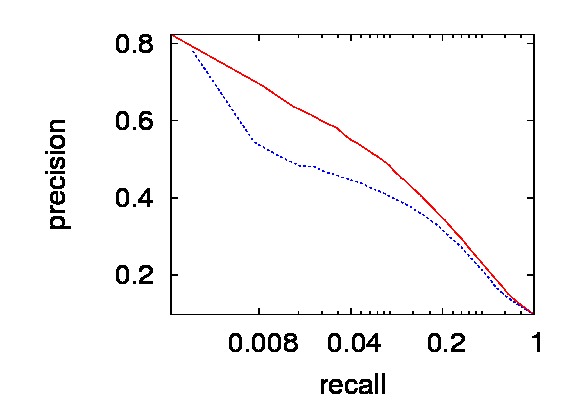

Supplement: Dataset S2 — Precision-recall figures for each tissue-specific network (red) versus the global (blue) network. (ZIP) [file pcbi.1002694.s002.zip › individual_figure/MA_0000581.txt.jpg]

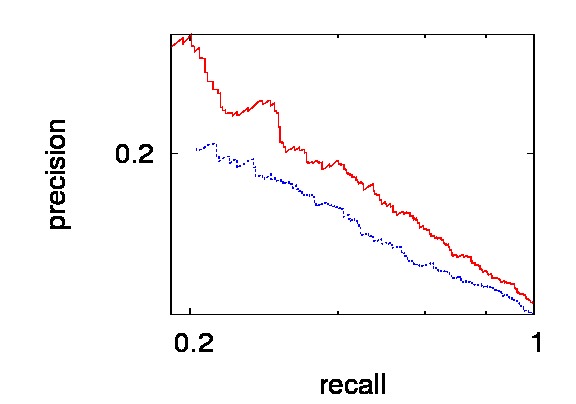

Supplement: Dataset S2 — Precision-recall figures for each tissue-specific network (red) versus the global (blue) network. (ZIP) [file pcbi.1002694.s002.zip › individual_figure/MA_0000997.txt.jpg]

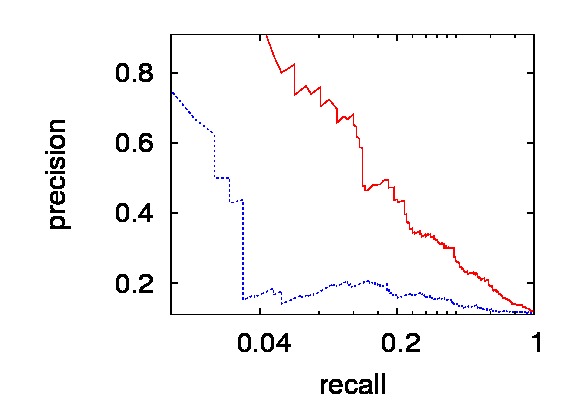

Supplement: Dataset S2 — Precision-recall figures for each tissue-specific network (red) versus the global (blue) network. (ZIP) [file pcbi.1002694.s002.zip › individual_figure/MA_0001080.txt.jpg]

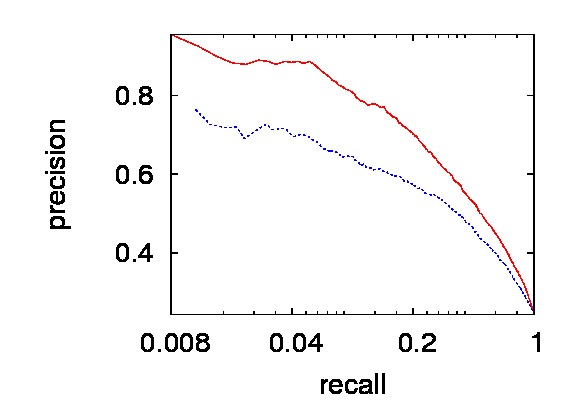

Supplement: Dataset S2 — Precision-recall figures for each tissue-specific network (red) versus the global (blue) network. (ZIP) [file pcbi.1002694.s002.zip › individual_figure/MA_0001273.txt.jpg]

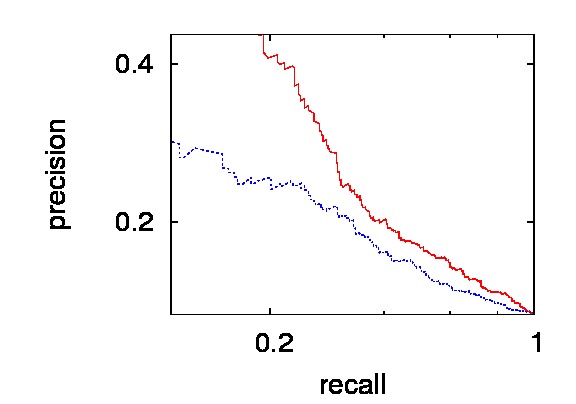

Supplement: Dataset S2 — Precision-recall figures for each tissue-specific network (red) versus the global (blue) network. (ZIP) [file pcbi.1002694.s002.zip › individual_figure/MA_0001319.txt.jpg]

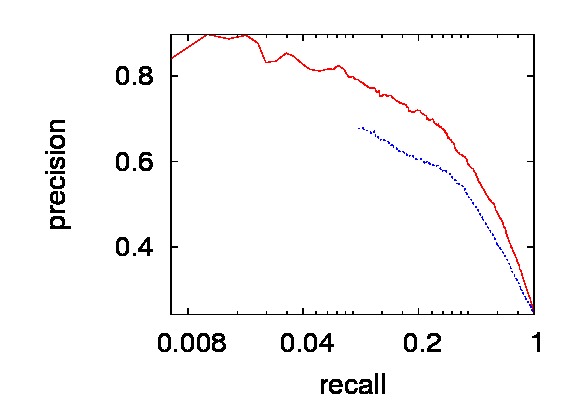

Supplement: Dataset S2 — Precision-recall figures for each tissue-specific network (red) versus the global (blue) network. (ZIP) [file pcbi.1002694.s002.zip › individual_figure/MA_0001359.txt.jpg]

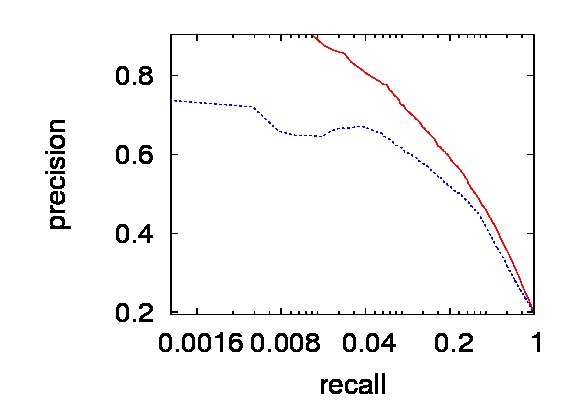

Supplement: Dataset S2 — Precision-recall figures for each tissue-specific network (red) versus the global (blue) network. (ZIP) [file pcbi.1002694.s002.zip › individual_figure/MA_0001459.txt.jpg]

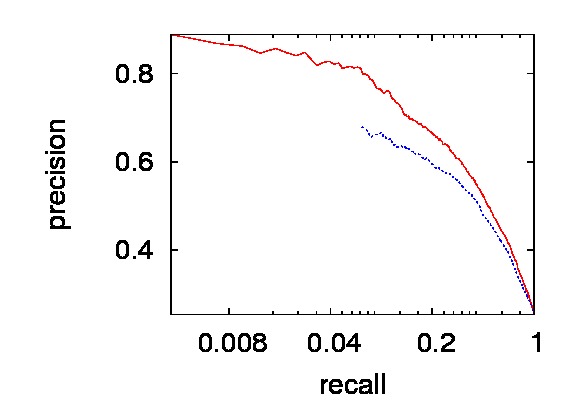

Supplement: Dataset S2 — Precision-recall figures for each tissue-specific network (red) versus the global (blue) network. (ZIP) [file pcbi.1002694.s002.zip › individual_figure/MA_0001541.txt.jpg]

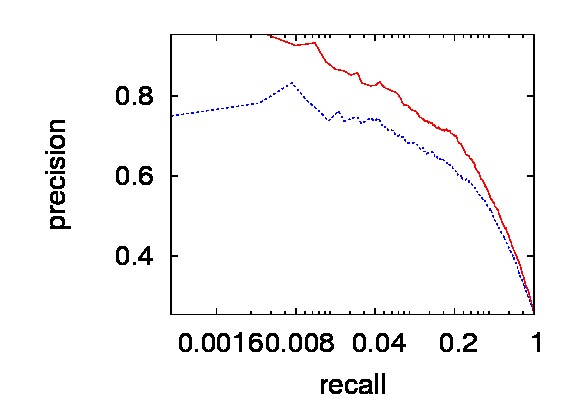

Supplement: Dataset S2 — Precision-recall figures for each tissue-specific network (red) versus the global (blue) network. (ZIP) [file pcbi.1002694.s002.zip › individual_figure/MA_0001542.txt.jpg]

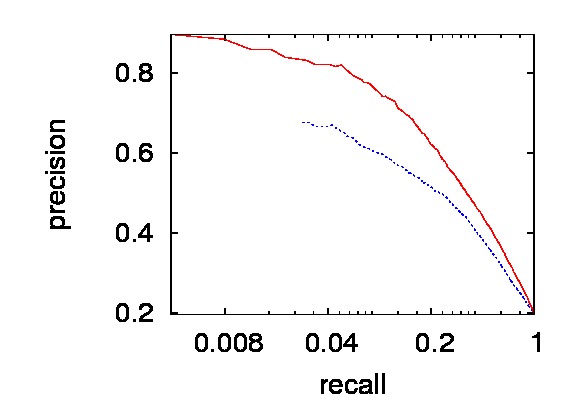

Supplement: Dataset S2 — Precision-recall figures for each tissue-specific network (red) versus the global (blue) network. (ZIP) [file pcbi.1002694.s002.zip › individual_figure/MA_0001910.txt.jpg]

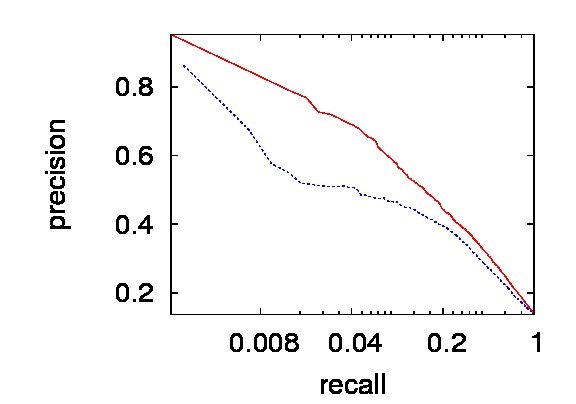

Supplement: Dataset S2 — Precision-recall figures for each tissue-specific network (red) versus the global (blue) network. (ZIP) [file pcbi.1002694.s002.zip › individual_figure/MA_0002413.txt.jpg]

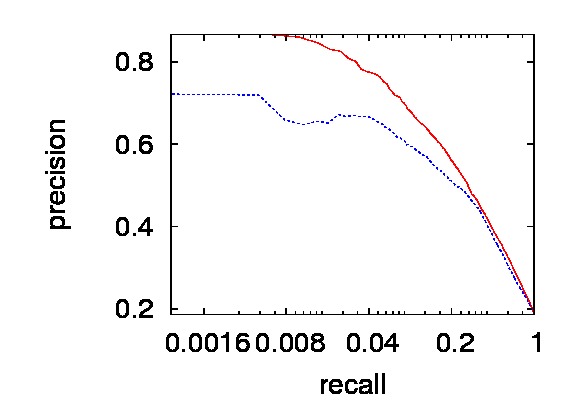

Supplement: Dataset S2 — Precision-recall figures for each tissue-specific network (red) versus the global (blue) network. (ZIP) [file pcbi.1002694.s002.zip › individual_figure/MA_0002418.txt.jpg]

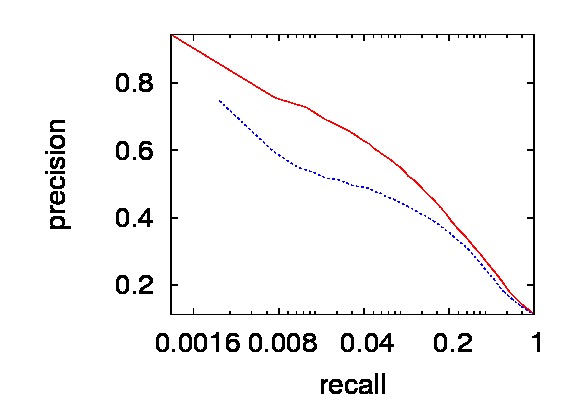

Supplement: Dataset S2 — Precision-recall figures for each tissue-specific network (red) versus the global (blue) network. (ZIP) [file pcbi.1002694.s002.zip › individual_figure/MA_0002431.txt.jpg]

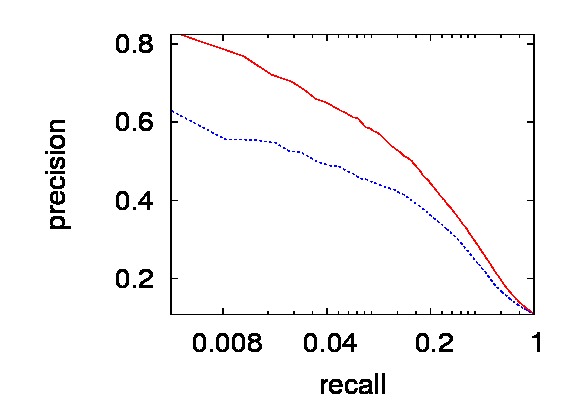

Supplement: Dataset S2 — Precision-recall figures for each tissue-specific network (red) versus the global (blue) network. (ZIP) [file pcbi.1002694.s002.zip › individual_figure/MA_0002434.txt.jpg]

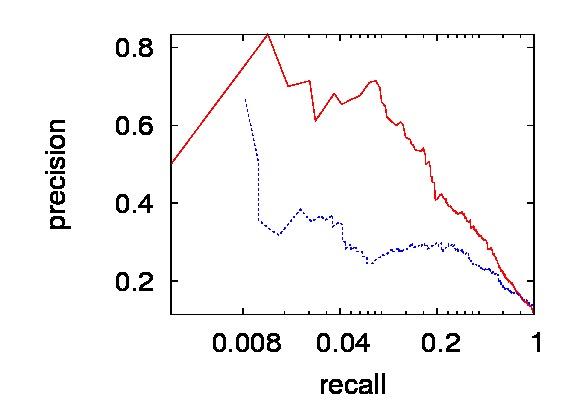

Supplement: Dataset S2 — Precision-recall figures for each tissue-specific network (red) versus the global (blue) network. (ZIP) [file pcbi.1002694.s002.zip › individual_figure/MA_0002435.txt.jpg]

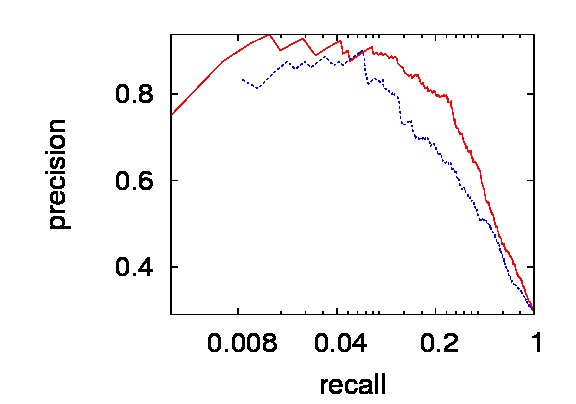

Supplement: Dataset S2 — Precision-recall figures for each tissue-specific network (red) versus the global (blue) network. (ZIP) [file pcbi.1002694.s002.zip › individual_figure/MA_0002443.txt.jpg]

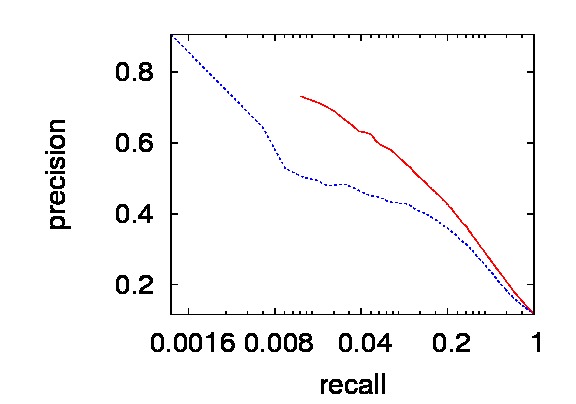

Supplement: Dataset S2 — Precision-recall figures for each tissue-specific network (red) versus the global (blue) network. (ZIP) [file pcbi.1002694.s002.zip › individual_figure/MA_0002444.txt.jpg]

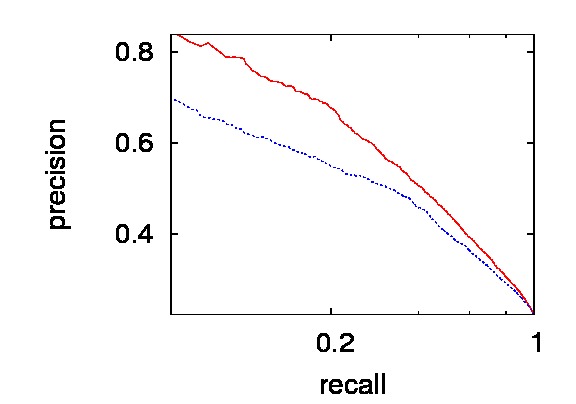

Supplement: Dataset S2 — Precision-recall figures for each tissue-specific network (red) versus the global (blue) network. (ZIP) [file pcbi.1002694.s002.zip › individual_figure/MA_0002446.txt.jpg]

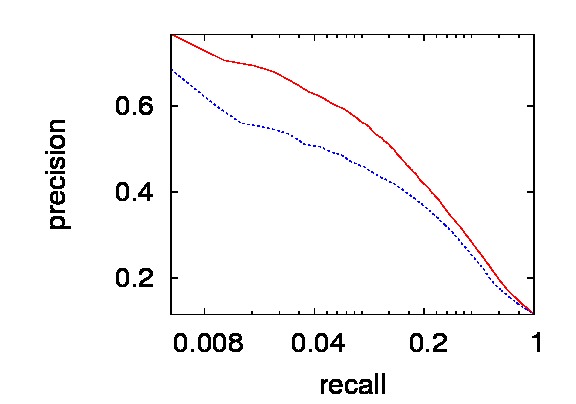

Supplement: Dataset S2 — Precision-recall figures for each tissue-specific network (red) versus the global (blue) network. (ZIP) [file pcbi.1002694.s002.zip › individual_figure/MA_0002449.txt.jpg]

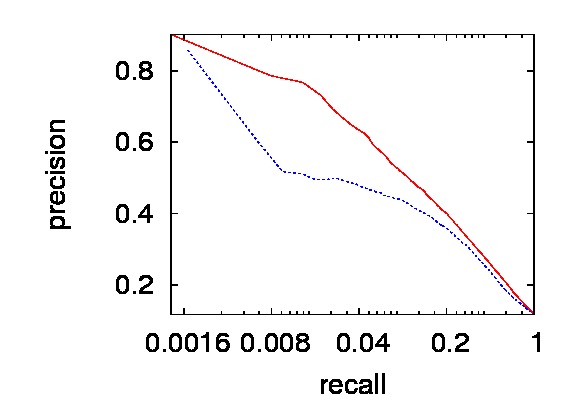

Supplement: Dataset S2 — Precision-recall figures for each tissue-specific network (red) versus the global (blue) network. (ZIP) [file pcbi.1002694.s002.zip › individual_figure/MA_0002473.txt.jpg]

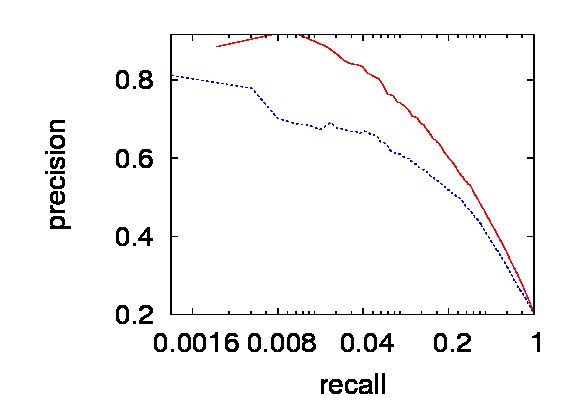

Supplement: Dataset S2 — Precision-recall figures for each tissue-specific network (red) versus the global (blue) network. (ZIP) [file pcbi.1002694.s002.zip › individual_figure/MA_0002474.txt.jpg]

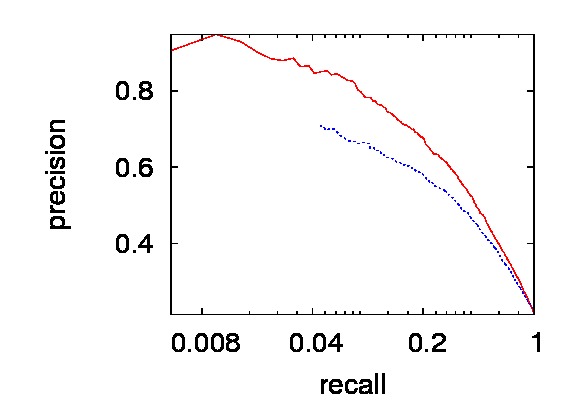

Supplement: Dataset S2 — Precision-recall figures for each tissue-specific network (red) versus the global (blue) network. (ZIP) [file pcbi.1002694.s002.zip › individual_figure/MA_0002636.txt.jpg]

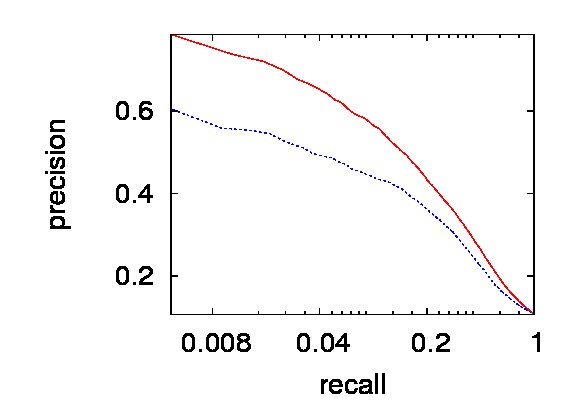

Supplement: Dataset S2 — Precision-recall figures for each tissue-specific network (red) versus the global (blue) network. (ZIP) [file pcbi.1002694.s002.zip › individual_figure/MA_0002711.txt.jpg]
